# Spatio-Temporal Control of Cell Adhesion: Toward Programmable Platforms to Manipulate Cell Functions and Fate

**DOI:** 10.3389/fbioe.2018.00190

**Published:** 2018-12-04

**Authors:** Chiara Cimmino, Lucia Rossano, Paolo Antonio Netti, Maurizio Ventre

**Affiliations:** ^1^Department of Chemical, Materials and Industrial Production Engineering, University of Naples Federico II, Naples, Italy; ^2^Center for Advanced Biomaterials for Healthcare@CRIB, Fondazione Istituto Italiano di Tecnologia, Naples, Italy

**Keywords:** dynamic platforms, cell adhesion, switchable signals, ligands, topographic, patterns, substrate stiffness

## Abstract

Biophysical and biochemical signals of material surfaces potently regulate cell functions and fate. In particular, micro- and nano-scale patterns of adhesion signals can finely elicit and affect a plethora of signaling pathways ultimately affecting gene expression, in a process known as mechanotransduction. Our fundamental understanding of cell-material signals interaction and reaction is based on static culturing platforms, i.e., substrates exhibiting signals whose configuration is time-invariant. However, cells *in-vivo* are exposed to arrays of biophysical and biochemical signals that change in time and space and the way cells integrate these might eventually dictate their behavior. Advancements in fabrication technologies and materials engineering, have recently enabled the development of culturing platforms able to display patterns of biochemical and biophysical signals whose features change in time and space in response to external stimuli and according to selected programmes. These dynamic devices proved to be particularly helpful in shedding light on how cells adapt to a dynamic microenvironment or integrate spatio-temporal variations of signals. In this work, we present the most relevant findings in the context of dynamic platforms for controlling cell functions and fate *in vitro*. We place emphasis on the technological aspects concerning the fabrication of platforms displaying micro- and nano-scale dynamic signals and on the physical-chemical stimuli necessary to actuate the spatio-temporal changes of the signal patterns. In particular, we illustrate strategies to encode material surfaces with dynamic ligands and patterns thereof, topographic relieves and mechanical properties. Additionally, we present the most effective, yet cytocompatible methods to actuate the spatio-temporal changes of the signals. We focus on cell reaction and response to dynamic changes of signal presentation. Finally, potential applications of this new generation of culturing systems for *in vitro* and *in vivo* applications, including regenerative medicine and cell conditioning are presented.

## Introduction

A major part of our understanding of cell biology is occupied by the effects of soluble signals (drugs, small molecules, peptides, proteins, growth factors) on cell behavior. In fact, several routes have been developed to formulate well-defined media that enabled studying cell response to specific soluble signals systematically (Yao and Asayama, 2017). There are comparatively less studies aimed at investigating the effects of insoluble matricellular signals, in the form of topographic relieves, patterns of biochemical moieties, or mechanical signals, on cell functions. These proved to dramatically affect cell behavior (Ventre et al., 2012). In different biomedical and clinical applications, cells are in close contact with material surfaces that can present one or more signals simultaneously. For instance, prosthetic devices or synthetic scaffolds invariably expose surfaces to the biological environment and the biochemical/biophysical characteristics of the surfaces can have a great impact on the implant performance *in vivo* (Williams and Bhatia, 2014; Rasouli et al., 2018). Therefore, achieving a sound knowledge on the role of material properties on cell functions would provide valuable elements to engineer devices with improved functions. This requires implementing design concepts and fabrication technologies that enable reproducing certain features of the extracellular matrix (ECM) that most effectively affect cell functions and fate. Advancements in materials engineering, functionalization methods and most importantly micro- and nano-fabrication technologies provided researchers with “artificial” alternatives to conventional rigid plates or glass, which more closely mimic the native microenvironment (Leijten and Khademhosseini, 2016). The integration of micro- and nano-engineered platforms with cell cultures not only allowed to elicit specific cellular reactions, thus controlling their functions and fates, but also enabled understanding cell-signal interactions. In fact, micro- and nano-engineered platforms display signals whose spatial arrangement may be targeted to the whole cell, subcellular compartments, cluster of receptors or even individual receptors, thus enabling to achieve a fine-tuning of a broad spectrum of signaling pathways (Dalby et al., 2014; Donnelly et al., 2018). In most of the cases, the signals displayed by materials are static in nature, i.e., once embossed on the culturing platform they cannot be changed in time and space. The native ECM is far from being a static repository of signals, as it constantly changes in time and space in response to or as a part of growth, aging, disease, injuries. For instance, temporal variations of the ECM, including changes in the microarchitecture and stiffness, play an important role in regulating different biological processes *in vivo* including differentiation and morphogenesis, but also the progression of pathologies (Lu et al., 2012; Handorf et al., 2015).

Cell biologists usually relied on reductionist approaches to study cell-signal interactions *in vitro* seeking systems aimed at reducing the complexity of interactions or at eliciting specific cell responses to investigate cell-signal interplay. These systems were instrumental to shape our understanding on the mechanisms underlying cell recognition and reaction to signals, but in most of the cases they are not able to capture specific aspects as multi signal stimulation or dynamic changes. This calls for novel platforms able to more closely mimic the ECM both in terms of signal display and dynamic changes of these signals.

Most of our knowledge on cell-material recognition and response to biochemical/biophysical signals arises from studies performed in two-dimensions (2D). Although most cells live in a three-dimension (3D) context *in vivo*, it is still debated whether adhesion in 3D is identical to a 2D environment or follows different routes (Harunaga and Yamada, 2011; Doyle and Yamada, 2016). This notwithstanding, we prefer to focus our attention on 2D systems as the development of dynamic materials is recent and mostly concerns planar surfaces. Examples of dynamic 3D environments have been already presented which showed enlightening results (Khetan et al., 2013; Das et al., 2016; Brown et al., 2018). However, achieving a precise control on the adhesive processes in 3D at a subcellular level is still challenging. Furthermore, 2D platforms should not be necessarily considered as pure artifacts. Specifically engineered materials proved to be very effective in controlling even complex aspects of cell behavior including stem cell self-renewal, targeted differentiation and morphogenesis (Nikkhah et al., 2012; Ventre et al., 2012; Ventre and Netti, 2016b). These examples have an intrinsic usefulness as the outcomes could be exploited in applications such as drug screening and discovery, cell-based therapies, and tissue engineering.

Increasing the complexity of material substrate to control cell functions and fate *in vitro* with the introduction of dynamically changing signals would better mimic a natural context thus enabling the possibility to guide and stimulate cells with improved effectiveness.

In this review we first illustrate the basic mechanism of cell ECM or material interactions focusing on cell adhesion processes to provide basic guidelines to engineer bioactive platforms to control cell behavior. We also discuss notable examples of cell interaction with “static” platforms to provide insights into cell's reactions and responses to specific signal arrangements, being more details on this aspect reported elsewhere (Bettinger et al., 2009; Ventre et al., 2012; Yao et al., 2013). The central part of the article reviews strategies and technologies to encode dynamic signals on material platforms. In particular, this work focuses on dynamic changes of ligands and their spatial patterns, micro- and submicro-scale topographies and material stiffness. Furthermore, emphasis is given to response of cells to the spatio-temporal changes of signal display. Finally, we will address limits of the current platforms and technologies suggesting possible ways to improve their performances thus creating systems that can affect cell functions in a more thorough and consistent manner.

## The Process of Cell Adhesion and Cell Response to Material Signals

**Figure 1:**
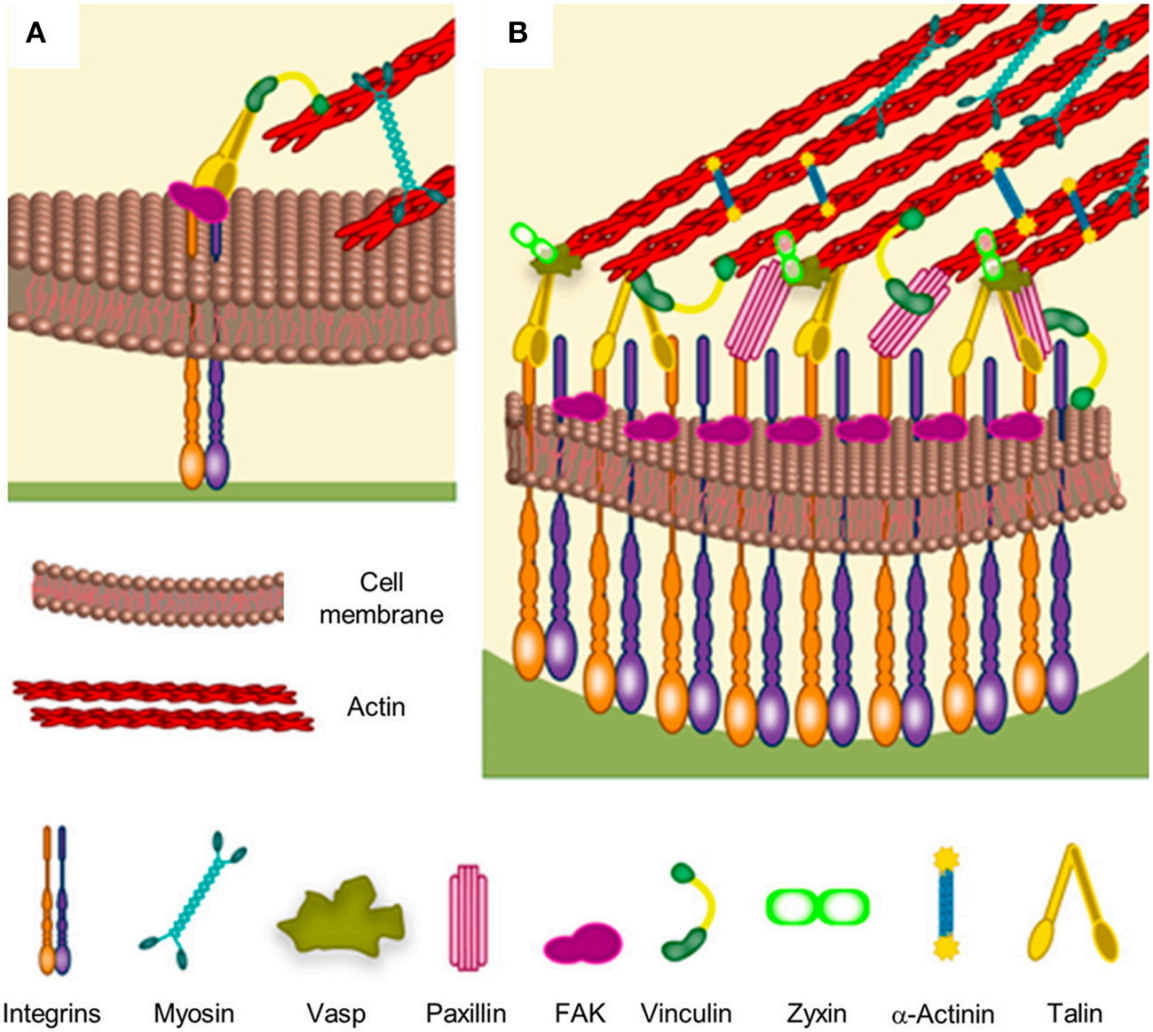
Schematic of focal adhesion formation and maturation. **(A)** Nascent adhesions are thought to form the binding of integrins to extracellular ligands. Additional cytoplasmic components, such as talin, FAK and paxillin are recruited to stabilize the ligand-bound clusters. Myosin-generated forces promote conformational changes of talin, which exposes binding sites for vinculin. Stable clusters can grow further by addition of other integrins and cytoplasmic molecules, thus generating a focal complex and then a focal adhesion. **(B)** The multi-layered structure of a mature focal adhesion comprises proteins with different functions: signaling (integrins, FAK, and paxillin), force transduction (vinculin and talin) and linkers to the cytoskeleton (Vasp, zyxin, and α-actinin). Reprinted with permission from Ventre and Netti (2016b). Copyright 2016 American Chemical Society.

Cells interact with the culturing microenvironment, including material surfaces, through an array of receptors that enable perceiving different chemical/physical cues such as roughness, hydrophobicity, ligand density and distribution, stiffness, and charge. The receptors involved in such a process of recognition are located on the cell membrane and they include immunoglobulin super family of cell adhesion molecules (IgCAMs), cadherins, integrins, selectins, and proteoglycans, as well as non-integrin collagen, laminin, and elastin receptors (Hinek, 1996; Campbell and Humphries, 2011; Humphries et al., 2015; Di Cio and Gautrot, 2016). In particular, the heterodimeric, transmembrane integrin receptors not only are responsible for initiating and maintaining stable adhesion, but take part in the formation of multiprotein signaling hubs that trigger biochemical events that greatly influence cellular behavior (Humphries et al., 2015). While non-specific interactions of cells with materials dominate early adhesion events, stable cell adhesions form when surfaces display ligands to which integrin receptors bind specifically (Cohen et al., 2004; Parsons et al., 2010). Integrins are constituted by α and β subunits. So far, 18 α and 8 β subunits have been identified that can combine to form 24 different receptors with different binding properties (Campbell and Humphries, 2011). Through different combinations of α and β subunits it is possible to determine the affinity of the heterodimer with specific ligands. Several aminoacidic sequences have been recognized to interact with integrins specifically. For example, IKLLI, LGTIPG, LRE, LRGDN, PDGSR, RGD, YIGSR, YKVAV in laminin, DGEA GFOGER and RGD in collagen I and KQAGDV, REDV, RGD, and PHSRN in fibronectin (FN). When sufficiently close to ligands, integrin dimers can acquire an activated form by undergoing to a conformational change that enhances the affinity to ligands and allows the interaction with proteins and signaling molecules from the cytoplasmic side (Calderwood, 2004). After activations, integrins cluster together in discrete locations of the membrane facing the adhesive surface. According to the availability of ligands and stability of the integrin-ligand complex, cluster may mature in focal complexes up to micrometric multiprotein entities named focal adhesions (FAs) (Figure 1). Proteins from the cytoplasmic side may stabilize the protein gathering and/or may act as a bridge with the actin cytoskeleton. Integrins, once activated, bind to actin fibers through cytoplasmic proteins such as α–actinin, talin, and vinculin. Cytoskeleton-generated contractile forces promote FA maturation through conformational change of mechanosensitive proteins that modify their functions. For example, vinculin undergoes to a conformational change upon force application, which improves the affinity toward other proteins involved in FA stabilization (Dumbauld et al., 2013). Also, talin possesses cryptic binding sites for vinculin that unfold and become available upon stretching (del Rio et al., 2009). Other examples of mechanosensitive proteins constituting the adhesion plaques are paxillin and p130cas (Janoštiak et al., 2014). Therefore, FAs constitute the gate through which the cytoskeleton interacts with the extracellular environment, allowing the perception of different signals, such as biochemical (ligands density and their spatial distribution) and biophysical (topography and mechanical characteristics), and the reaction to these is accompanied by alterations in FA features, cytoskeleton assembly and cell-generated forces (Geiger et al., 2009).

Moreover, molecules related to FAs are important signaling proteins such as kinases [focal adhesion kinase (FAK), Src] and GTPase (Rho, Rac) that alter their activities when subjected to the mechanical forces exerted by the actomyosin machinery. For example, contractile forces can induce the phosphorylation of additional sites of the Src family kinases substrate p130CAS, which can eventually trigger signal transduction pathways (Sawada et al., 2006). The conversion of mechanical stimuli (either externally applied or cell-generated) into intracellular biochemical events is usually referred to as mechanotransduction (Eyckmans et al., 2011). These mechanical signals are also perceived by the nucleus, as the actin cytoskeleton is connected directly to the nuclear membrane (Isermann and Lammerding, 2013). The nucleus reacts to the forces generated by actin by changing its shape, modifying the assembly of chromatin as well as the accessibility of enzymes and transcription factors, eventually altering gene expression (Li et al., 2011; Gupta et al., 2012; Jain et al., 2013). In this scenario elements affecting cell adhesion (ligand availability and patterning or the transmission of mechanical forces) through biochemical/biophysical signals embossed on material surfaces may enable the activation of intracellular biochemical events ultimately affecting cell behavior in a process that we refer to as the material-cytoskeleton crosstalk (Ventre et al., 2012).

A series of strategies has been developed to control biochemical/biophysical properties of the culturing materials that mostly affect FA formation and growth. The first examples are those concerning functionalization with ligands. Several proteins or peptides involved in cell adhesion process may be linked to the material surfaces by covalent binding or by physical interactions. Arginylglycylaspartic acid (RGD) is among the most studied and used ligand to control cell adhesion (Hersel et al., 2003; VandeVondele et al., 2003). Early studies aimed at identifying the optimal density of the ligands that enable cell adhesion and spreading. In this regard, Massia and Hubbell observed that fibroblasts spreading on glass surfaces required a minimum RGD density of 1.0 × 10^−15^ mol/cm^2^ (corresponding to an interligand spacing of ~440 nm), while a density of 1.0 × 10^−14^ mol/cm^2^ (interligand spacing of ~140 nm), was necessary to promote the formation of focal contacts and stress fibers (Massia and Hubbell, 1991). Therefore, precise spatial positioning of adhesive spots may have a profound impact on the process of cell adhesion. Micro- and nano-fabrication techniques have enabled the realization of cell adhesion substrates allowing to modulate the dimension, positioning and spacing of the adhesive zones on a micro- and submicro-scale. For example, block copolymer micelle nanolithography allows to control spacing and density of individual ligands. Through this method, it was proven that adhesion formation occurs in a specific range of interligand densities, i.e., from 58 to 73 nm. Ligand densities surpassing this range cause an excessive adhesion and a decrease of cellular mobility, while lower ligand densities do not allow cell spreading (Arnold et al., 2004). Exerting a tight control on individual FA localization and shape requires achieving a sharp adhesion mismatch on the surface, i.e., zones conducive for FA formation and growth juxtaposed to others not allowing integrin engagement. This prevents FAs to grow and acquire shapes uncontrollably. Surfaces exhibiting micro- or nano-scale relieves in the form of pillars or pits can reproduce such a scenario effectively. In fact, the formation of FAs strongly depends on the geometry of the topography. Proteins and ligands can adsorb non-uniformly or might not be readily available to cells thus impairing FA dynamics (Lord et al., 2010). There is still a lack of systematic studies that rigorously indicate which combinations of pattern height, spacing and size promote or impair cell adhesion. However, experimental evidences suggest that characteristic dimensions that most effectively interfere with FA dynamics exist, which are suggestive of a possible common mechanism of cellular response to topographies. Examples of insufficient cell adhesion have been reported when cells are seeded on nanoprotrusions with a feature height above 80 nm, as this dimension hampers integrin clustering (Lim et al., 2005b; Biggs et al., 2010). The same situation of insufficient adhesion occurs when cells are grown on pillars whose diameters are <70 nm and the interpillar spacing is >300 nm. FAs formation takes place when integrins are able to bridge the gap between neighboring features or when the distance between these is ~70 nm (Kim et al., 2005; Lee et al., 2008; Sjöström et al., 2009). Other studies showed that the pitch and size of nanopits are also fundamental parameters in governing cell adhesion. For instance, FAs growth is confined in the interpit area, thus limiting the growth on arrays of nanopits of 100 nm in depth, 120 nm in diameter and 300 nm in spacing arranged in ordered square or hexagonal lattices (Biggs et al., 2007; Dalby et al., 2007).

The geometry of the topographic model, besides influencing the formation of adhesions, strongly affects FAs orientation. In fact, while pillars and pits arrays limit FAs elongation, micro- and nano-gratings guide FAs growth in a specific direction. Directional growth of FAs strongly affects cytoskeleton assembly, as stress fibers are oriented in the same direction of the underlying pattern. In this way the entire cell body assumes an elongated morphology and migrates along the pattern direction. This phenomenon is known as “contact guidance” (Teixeira, 2003; Ventre et al., 2014). This, however, depends on the size and spacing of the topographic features as too wide or too close ridges do not exert an effective confinement on FAs, thus impairing their guiding effect. For example, osteoblasts cultivated on nanogratings with ridge spacing <75 nm allowed integrin clustering over the grooves that caused the cells to respond as on flat substrates (Lamers et al., 2010). Conversely, a change in osteoblast response was observed when lateral spacing exceeded 75 nm. Cells were elongated and coaligned to the pattern direction with ridges and grooves of 80 nm in width or on patterns with 50 nm ridges and 100 nm grooves. Instead, no significant orientation was observed on the pattern with 100 nm ridges and 50 nm grooves (Lamers et al., 2010). In order to visualize the dynamic interplay between FA formation/maturation and nanogrooved patterns, Natale et al. transfected preosteoblasts with plasmids characterized by fluorescent actin and paxillin (Natale et al., 2014). Actin fibers were observed to bundle and align in directions, not necessarily parallel to the pattern direction. However, fibers oriented in directions other than that of the pattern ended with dashed adhesions that rapidly collapsed under the effect of actin-generated forces. In contrast, the FAs that grew along the direction of the pattern appeared more stable and promoted the formation of actin bundles parallel to the ridges.

The stiffness of the culturing substrate profoundly affects FA morphology and stability (Pelham and Wang, 1997). Usually, cells on rigid materials (plastics, highly crosslinked elastomers, stiff hydrogels) express long and wide FAs connected to well-defined actin bundles. Conversely, cells on compliant substrates (scarcely crosslinked elastomers or soft hydrogels) possess punctuate and highly dynamic FAs and a destabilized cytoskeleton (Ventre and Netti, 2016b). The molecular mechanisms underpinning cell recognition of stiffness are not thoroughly clear as some evidence suggests that different stiffnesses lead to differences in anchoring densities of proteins thus interfering with sensing, whereas other authors report that cells react to changes in stiffness independently from ligand anchoring (Trappmann et al., 2012; Wen et al., 2014). However, the extent of contractility generated on either soft or stiff substrates differently activates mechanotransduction events at FA or nuclear level, ultimately affecting cell functions and fate (Engler et al., 2006; Anderson et al., 2016).

Surface engineering has benefitted from these observations because the material characteristics may be modified in order to influence the dynamic formation and maturation of the FAs hence affecting the activity of the signaling proteins or altering the structure and contractility of the cytoskeleton. Today, concepts as ligand density and patterning, topography and roughness, or material stiffness are regarded as crucial players in affecting various aspects of cell behavior ad are increasingly taken into consideration when designing experiments aimed at capturing certain features of the ECM. However, as the response to soluble drugs or factors depends on their dose and temporal delivery, it is clear that the static presentation of biochemical/biophysical material signals does not fully mimic the dynamic nature of the native microenvironment and might lead to abnormal or non-physiologic reactions. This calls for novel culturing systems conceived to display biochemical/biophysical material signals according to predefined spatio-temporal programmes.

## General Concepts on Dynamic Substrate Engineering

Synthetic materials with properties that are static in time are often inadequate for mimicking natural cell environments, where temporal changes in chemical and physical properties are important in a wide variety of contexts including development, differentiation, morphogenesis, diseases progression, wound healing, and homeostasis (Frantz et al., 2010; Lu et al., 2011; Bonnans et al., 2014; Kular et al., 2014). To assess the role of the dynamic changes of the biochemical/biophysical microenvironment on cell behavior, the development of platforms enabling a fine control on the spatio-temporal presentation of adhesion sites is key central. Recent developments in materials engineering have led to a variety of approaches in which material properties can be dynamically and reversibly modified in response to user-directed stimuli such as light, temperature, pH, or other external fields (Roy et al., 2010; Stuart et al., 2010). Concerning the fabrication of responsive biomaterials for dynamic signal presentation, different chemical/physical characteristics of the substrate, as well as those of the external stimulus, must fulfill specific requirements. Responsive materials may contain moieties responsible for endowing the materials with the dynamic behavior (Liu and Urban, 2010). These moieties do not have to harm cells whether inactive or activated by an external stimulus. Furthermore, the latter does not have to hamper cell viability by itself or does not have to cause leaching of toxic substances from the material. For instance, temperature or pH responsive materials should perform the intended transformations in a limited range of temperature or pH values (Watanabe and Okada, 1967; Kruse et al., 2017). Similarly, light can only be used at specific wavelengths and intensities (Masuma et al., 2013). Additionally, the choice of employing a particular stimulus may be dictated on whether a uniform or local material actuation is necessary. For instance, temperature and electric fields can be suitable for activating substrates uniformly, whereas laser beam can act locally. This can enable dynamic exposure to signals at a cellular or subcellular level. However, additional devices, such as UV masks or electrodes, can be employed to force the external field to act locally rather than uniformly. Alternatively, the responsive moieties could be spatially patterned thus causing the culturing platforms to be responsive in selected regions only (*vide infra*). Literature is sometimes ambiguous on the term static and dynamic (referred to signal presentation). We here refer to dynamic presentation when signals and more specifically adhesive signals significantly change configuration and or location in a way that cells recognize such a change and modify their behavior accordingly. The extent of change in signal display depends on the specific context that has to be analyzed as changes may occur on different length or time scales (receptor-whole cell level; seconds to weeks).

In what follows we present consolidated strategies along with the recent developments for engineering synthetic substrates displaying signals dynamically. More specifically, we will focus our attention to those signals directly affecting cell adhesion events at different length scales: from FA to whole cell level. We will therefore emphasize the role of dynamic display of biochemical (ligands), topographic and mechanical signals on cell adhesion, spreading, migration, and differentiation.

## Dynamic Display of Ligands

Self-assembled monolayers (SAMs) are one among the first and perhaps most used and studied platforms to enable signal display dynamically. SAMs can be defined as orderly assemblies of molecules formed by the adsorption of an active surfactant on a solid surface (Ulman, 1996). The molecular structure of the individual building blocks that form SAMs can be divided in three parts: a head that binds to a substrate (generally a metal); a central part acting as spacer, whose chemical properties determine the interactions with the surrounding molecules, ultimately defining the packing and stability of the monolayer, which is usually achieved by van der Waals attractive forces between adjacent chains or by the introduction of specific intermolecular interactions; a tail group which can be inert, bioactive or susceptible to functionalization (Ulman, 1996; Srisombat et al., 2011). The composition of SAM-based platforms can be predetermined and the chemical properties can be considered uniform over relatively large areas. Furthermore, SAMs are prone to additional functionalization or patterning after they are formed (Mrksich, 2009). These are fundamental requirements to fabricate artificial platforms to study cell adhesion events or cell response to specific arrangements of ligands in a systematic manner. Examples of SAMs used as cell culture substrates are thiol or silane head groups derivatives on gold or silicon, respectively (Schreiber, 2004; Onclin et al., 2005). Oligo(ethylene glycol) SAMs were used as non-fouling protein surfaces and consequently cell repellent substrates. Alternatively, a number of biomolecules, including DNA, RGD, Laminin-derived Peptide PA22-2, FN, Vascular Endothelial Growth Factor, have been used to bioactivate SAM surfaces on active groups present on the molecules tail (Bamdad, 1998; Liu et al., 2007; Mendes, 2008; Jans et al., 2009; Mrksich, 2009; Afara et al., 2012).

Another method to fabricate cell culturing substrates is polymer casting. Surfaces of cast polymer are compatible with functionalization techniques such as chemical grafting or topographic embossing (Vendra et al., 2011). Phase separation of polymers is a fast and reliable method that enables controlling the morphology and dimensions of micro- and nano- domains in the polymer, which affect surface topography and protein adsorption thus impacting on cell adhesion and eventually cell fate (Lim et al., 2005a; Krishnamoorthy et al., 2006; Frith et al., 2012).

The synthesis of polymer brushes, i.e., thin coatings made of polymer chains covalently attached to a substrate, represents a versatile method to fabricate controlled environments for cell cultures (Chen et al., 2017). Chains can be attached directly to reacting substrates (grafting-to) or might be allowed to polymerize from surface anchored initiators (grafting-from). In both cases, the density of polymers should be sufficiently high to force polymer chains to be in a stretched form, preventing chain collapse in a random coil form. Of the two methods, grafting-from produces high density brushes as preformed polymers in the grafting-to method can be displaced apart owing to steric hindrance (Advincula, 2004). Several parameters related to polymer brush processing including, chain architecture and length, density, functionalization, all proved to significantly affect cell adhesion and behavior (Krishnamoorthy et al., 2014). The development of new synthetic routes allowed to tune these properties, thus leading to the fabrication of culturing platforms with tailored signal display (Chen et al., 2017; Feng and Huang, 2018).

Besides solid substrates, the functionalization of hydrogel surfaces has been successfully used to control local chemical/physical properties of the culturing microenvironment (DeForest and Anseth, 2012; Guvendiren and Burdick, 2013a). Both synthetic and natural hydrogels emerged as versatile and promising system for cell culture since they mimic important aspects of the native ECM such as mechanical properties, viscoelasticity, porosity, along with their ability to sequester proteins and growth factors (Caliari and Burdick, 2016). From a practical standpoint, macromolecules constituting hydrogels can be extensively modified (in pre- or post-processing) thus allowing to tailor the chemical, physical properties of the network in an orthogonal manner and to endow the substrate with complex functions (Kharkar et al., 2013; Ventre and Netti, 2016a). Hydrogels find their natural application field as 3D cell culturing systems. However, they are also useful to study certain cell behaviors in 2D such as mechanosensing and mechanotransduction that require substrate stiffness to be finely tuned (Thiele et al., 2014).

### Electrically Controlled Presentation of Ligands

**Figure 2:**
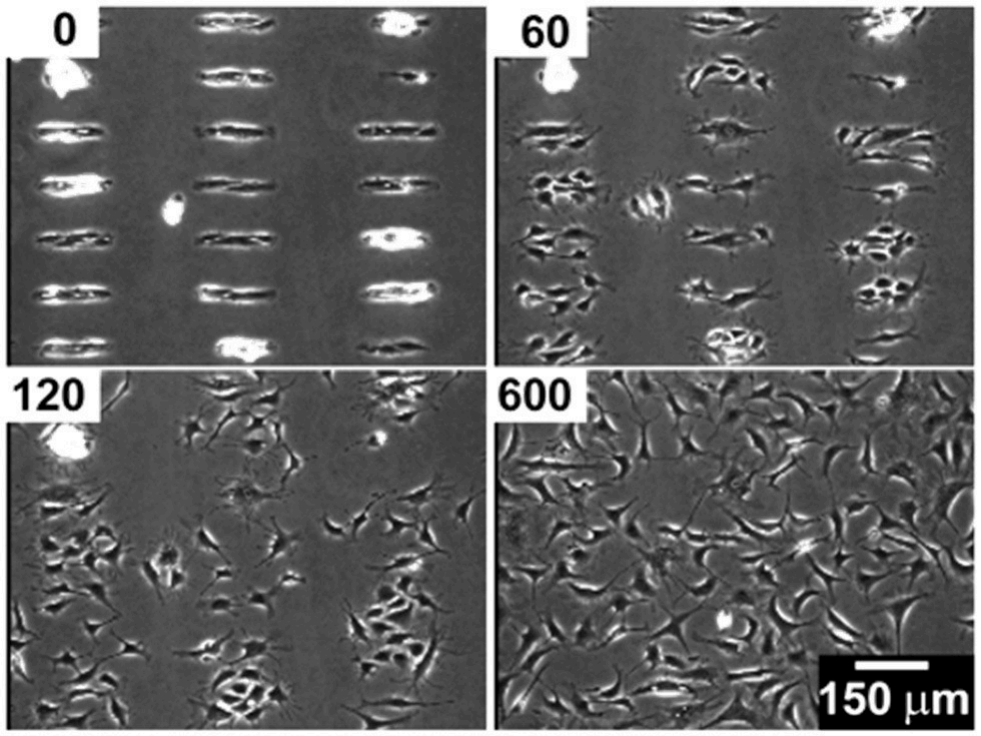
Bovine capillary endothelial cells adhered on a micropatterned gold substrate backfilled with a cell repellent ethylene glycol-terminated SAMs. Application of a −1.2 V voltage pulse (30 s) caused SAMs desorption and subsequent protein adsorption on the bare gold. This enabled cells attachment and migration on previously inert areas. Top left insets indicate the minutes elapsed after the voltage pulse. Reprinted with permission from Jiang et al. (2003). Copyright 2003 American Chemical Society.

**Figure 3:**
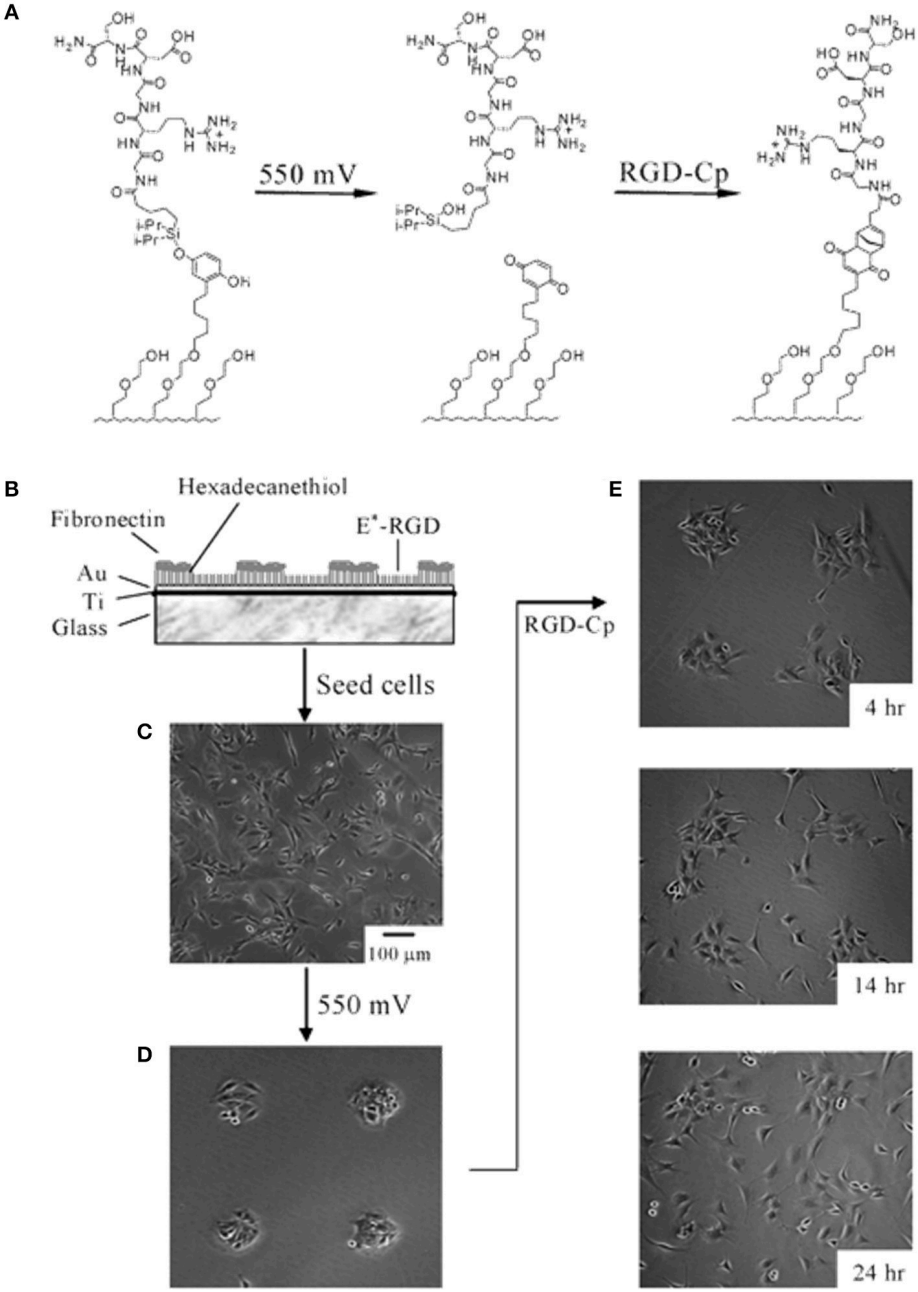
Electrochemical release of ligands. **(A)** A SAM presenting the O-silyl hydroquinone when subjected to a 550 mV voltage oxidizes to benzoquinone, with hydrolysis of the silyl ether and release of the RGD ligand. The resulting benzoquinone may react with soluble cyclopentadiene-tagged RGD via a Diels-Alder reaction, which immobilizes the second ligand. **(B)** Illustration of the dynamic substrate combining the electrochemical release of RGD ligands and of the cells with the (secondary) immobilization of RGD ligands. A SAM was patterned into FN coated circular regions surrounded by RGD tethered with an electroactive linker (E^*^-RGD). **(C)** Swiss 3T3 fibroblast cells adhered to both FN and E^*^-RGD over the SAM substrate. **(D)** An electrical potential of 550 mV applied to the gold substrate for 5 min caused RDG and cell release, whereas cells on FN patterns remained attached. **(E)** Supplementation with soluble cyclopentadiene-tagged RGD caused the secondary immobilization of the ligand restoring cell adhesion and migration. Adapted with permission from Yeo et al. (2003). Copyright 2003 American Chemical Society.

**Figure 4:**
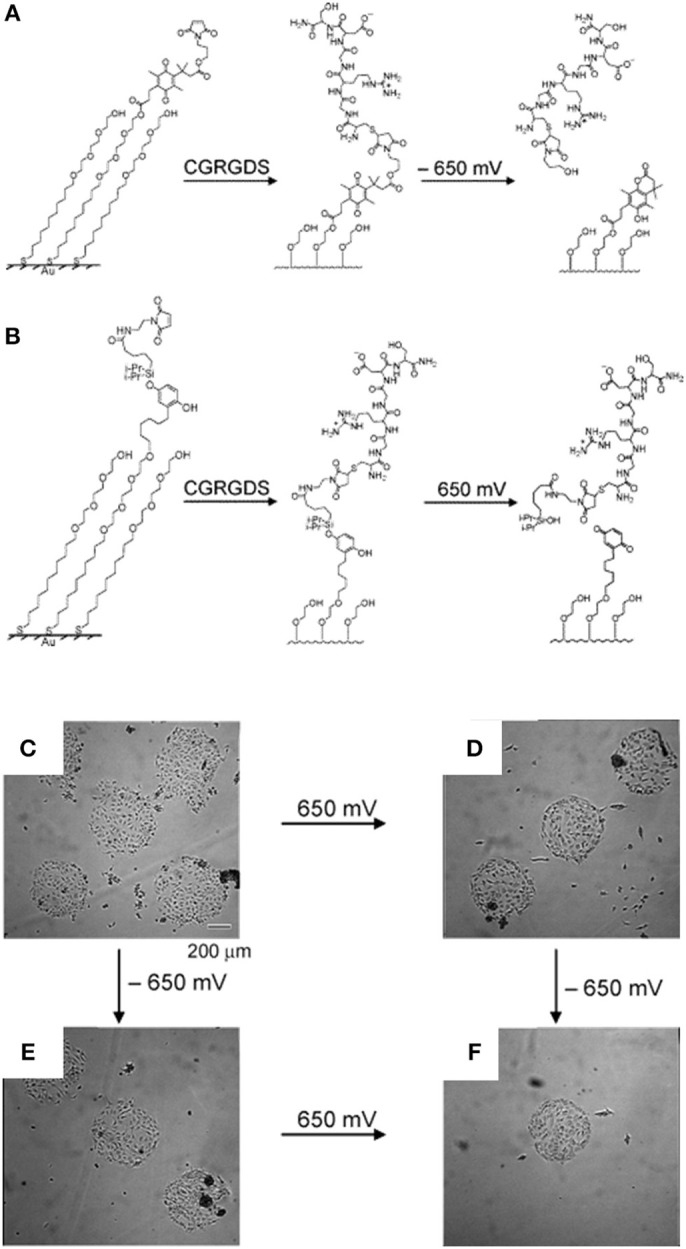
Dynamic substrates for the selective release of ligands in response to applied potentials. **(A)** A SAM presenting a RGD ligand tethered to an electroactive quinone ester. Upon application of the negative voltage causes reduction of the quinone to the corresponding hydroquinone, a cyclization reaction gives a lactone with the release of the RGD ligand. **(B)** A SAM presenting a RGD ligand tethered to an electroactive O-silyl hydroquinone. Application of the positive voltage causes electrochemical oxidation to give a benzoquinone, with the hydrolysis of the silyl ether and the ligands. Effects of the selective electrochemical release of ligands on cell adhesion. **(C)** Swiss 3T3 fibroblasts adhere to circular patterns containing either electroactive O-silyl hydroquinone or quinone ester. **(D)** After application of 650 mV potential, cells detached only from regions presenting the electroactive O-silyl hydroquinone. **(E)** The application of −650 mV potential caused cell release of cells from the regions presenting the electroactive quinone ester only. **(F)** The subsequent application of a potential of −650 mV **(D)** or 650 mV **(E)** results in an additional release of cells. Reprinted (adapted) with permission from Yeo and Mrksich (2006). Copyright 2006 American Chemical Society.

Although SAMs were originally intended as static surfaces, the incorporation of moieties in the polymer chains that induce conformation changes, bond breaking or react with other species under external stimuli, endows SAM-based surfaces with dynamic behavior. In a seminal work by Jiang et al. electrical desorption of SAMs was used to fabricate dynamic surfaces to study bovine capillary endothelial cell migration (Jiang et al., 2003). Micro-printed patters of protein repellent alkanethiols [HS(CH_2_)_11_(OCH_2_OCH_2_)_3_OH and HS(CH_2_)_17_CH_3_] on gold confined cells in specifically designed rectangles. The application of a cathodic voltage pulse (−1.2 V) caused the alkanethiols to desorb. In the absence of a protein repellent layer, soluble FN in the medium readily adsorbed on the bare gold enabling the cells to move from the initial rectangle onto the newly available adhesive regions (Figure 2). Authors from the same research group exploited this strategy to investigate the effect of cell shape on migration and in particular on the initial direction of displacement (Jiang et al., 2005). The authors printed via microcontact printing (μCP) adhesive islets of different shapes (either symmetric circles, rectangles, squares or asymmetric teardrops, wide drops, narrow drops, triangles). By analyzing the Golgi apparatus, centromere and cell centroid positioning the authors noticed that cells had a marked tendency to displace toward the blunt end of the drop-like shapes. The authors suggested that the shape asymmetry was sufficient to bias the direction of motion. The combination of μCP and electrically switchable SAMs, makes this method a robust platform that enables studying cell dynamics without possible complications arising from cell-cell contacts. However, once SAMs desorb, adhesion patterns cannot be restored. An attempt to integrate electric signal switch and reversibility was proposed by Yeo et al. who fabricated hexadecanethiol-based SAMs on gold incorporating O-silyl hydroquinone moiety functionalized with RGD (Yeo et al., 2003). The application of an electric potential to the substrate causes the oxidation of the O-silyl hydroquinone group yielding the corresponding benzoquinone with the hydrolysis of the silyl ether and the release of the RGD (Figure 3A). To prove the validity of the proposed scheme, the authors micropatterned SAMs in the form of 220 μm discs of hexadecanethiol on gold and the remaining regions were filled with a monolayer of RGD electroactive alkanethiolate as described above (Figure 3B). The system was coated with FN, which favored homogeneous adhesion of Swiss 3T3 (Figure 3C). The application of an electric potential of 550 mV released the RGD, thus promoting cell detachment. Only the cells on the non-electroactive patterned discs remained in place (Figure 3D). RGD functionalized cyclopentadiene moiety was then supplemented to the culture medium; this reacted with the benzoquinone tails by means of a Diels-Alder reaction. Microscopic observations revealed cells that invaded the newly formed RGD activated regions (Figure 3E). The authors showed an on-off-on pattern of signal display; this, however, required additional steps involving the introduction of specifically synthesized chemicals. A further development of this scheme involved the use of either electroactive RGD tagged quinone ester or the conventional O-silyl hydroquinone (Yeo and Mrksich, 2006). The former releases the RGD under reductive potentials, as opposed to the latter that is sensitive to oxidative potentials (Figures 4A,B). To prove selective release of fibroblasts from the substrate the authors micropatterned circular SAMs on gold with either of the two electroactive alkanethiolates all of them displaying RGD. Swiss 3T3 fibroblasts adhered on the micropatterned regions only (Figure 4C). Applying either a negative or positive potential caused selective detachment of cells from the electroactive quinone ester or O-silyl hydroquinone moieties, respectively (Figure 4D–F). These studies demonstrate the effectiveness of using small voltages to trigger dynamic surfaces. Furthermore, the electric potentials applied are compatible to cell culturing conditions.

A possible drawback of electric desorption of SAMs consists in the impossibility to change the electric potential locally thus achieving a spatial control on the desorption, unless networks of electrodes are assembled together (Yoon and Mofrad, 2011). A possible solution to this issue was proposed by Ng et al. who extended the concept of electrically switching surfaces (Ng et al., 2012). Rather than controlling chemical reactions with electric potentials the authors fabricated charged hexa(ethylene glycol) based SAMs on a silicon electrode. Charged moieties (either sulfonate or ammonium) were conjugated at the distal end of the SAM chains that were juxtaposed to chains terminating with GRGDS adhesive peptide. When the system was subjected to an electric potential of the same polarity as the charged moiety, then polymeric chains projected out masking the neighboring ligands. Conversely, reversing polarity caused the chain to fold back, leaving exposed and accessible the ligands. The authors also created patterns of cationic and anionic regions and were able to revert cell-adhesive/cell-repellent zones dynamically and reversibly.

### Photo-Controlled Presentation of Ligands

The use of light as a possible trigger for dynamic surfaces has attracted considerable interests since it in principle allows to directly implement spatial patterns of “switched on-off” signals by controlling the exposure of the irradiating light with (for example) a photomask. This is a substantial advantage as opposed to electrical controlled surfaces for which case embossing adhesive patterns of signals could require the design of complex networks of electrodes or additional manipulations of the substrate with, for instance μCP.

Using UV irradiation of a conventional microscope, Nakanishi et al. were able to emboss microscale patterns on alkylsiloxane based SAMs on glass, displaying a photocleavable 2-nitrobenzyl tail group (Nakanishi et al., 2006). Coating the photocleavable SAM with bovine serum albumin (BSA) prevented cell adhesion. Conversely, exposure to a 365 nm UV radiation caused tail cleavage, BSA release and exposure of OH groups. Fibronectin solubilized in the medium adsorbed on the photocleaved regions enabling adhesion. UV exposure can be performed directly, thus activating a wide area or through a photomask. In this case, microscale features could be formed enabling to study cell dynamics at a single or even at a subcellular level. In particular, HEK293 cells on a square array of 6 μm^2^ adhesive islets formed FAs on the patterned islets only, whereas they were able to stretch and spread across the non-adhesive areas. These data confirm that photomasking enables achieving sufficient spatial resolution to study cell adhesion at a FA level dynamically (Nakanishi et al., 2006). The same group exploited a similar technique to study cell migration and membrane extension on the dynamic surfaces (Nakanishi et al., 2007). In this case, Pluronic 108 was used as cell repellent backfill. NIH3T3 cells were first seeded on 25 × 25 μm^2^ squares and then UV light was directed in order to form either a 25 or 5 μm wide stripe protruding out the edge of the original square. In the case of square patterns, cells spread over the entire new bioadhesive area, displaying actin bundles with FA oriented at various angles at their termini. Conversely, NIH3T3 cells displayed long protrusion along the narrow stripe with coaligned FAs and actin bundles. Photoactivated desorption of cell repellent compounds possesses the great advantage of allowing a greater level of spatial control of the adhesive properties of the substrate. Cellular and subcellular resolution can be achieved using conventional apparatuses. However, the methods illustrated so far still rely on the non-specific adsorption of adhesion moieties from the medium which could be susceptible to cell-mediated remodeling.

In order to control cell-material adhesion at the molecular level directly and irrespective of influences arising from material properties Petersen et al. exploited a photosensitive ligand caging strategy (Petersen et al., 2008). The authors synthesized a cyclo(-Arg-Gly-Asp-D-Phe-Val) c-(RGDfK), which shows improved affinity for the α_V_β_3_ integrins, protected with a photolabile 3-(4,5-dimethoxy-2-nitrophenyl)-2-butyl ester attached on the Asp residue of the ligand. A tetra(ethylene glycol) linker was anchored to a silica surface from the one side, whereas the caged ligand was on the tail side. 3T3 fibroblasts were seeded on the substrate before and after irradiation with UV at 351 nm. The authors found more than 10-fold increase in adhered cells on substrates exhibiting the ligand in the active form. Furthermore, the authors used photomasks to activate the substrate on selected regions and, as expected, cells adhered on the exposed ligands predominantly. However, a degree of non-specific adhesion was observed on non-irradiated areas especially at longer culturing times.

**Figure 5:**
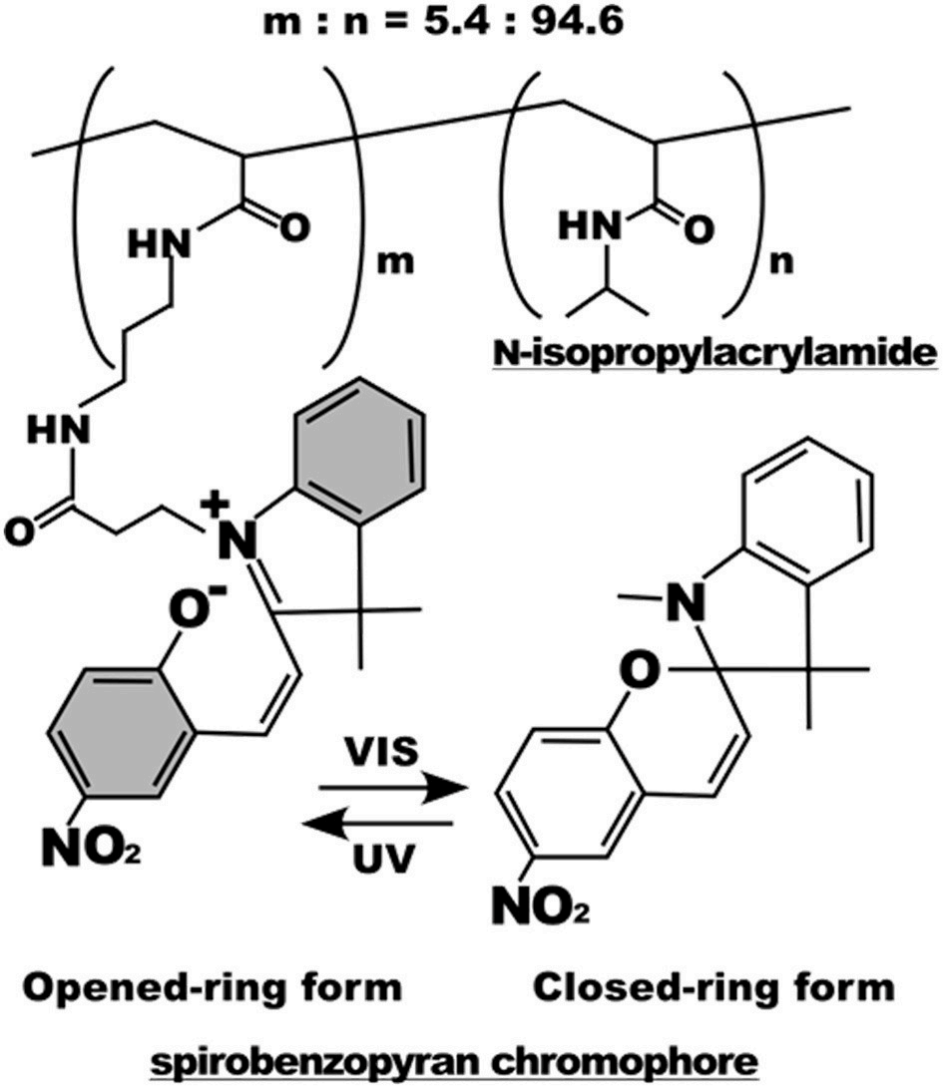
Chemical structure of the poly(N-isopropylacrylamide) with nitrospiropyran chromophore sidechains. Below, photoisomerization scheme of the nitrospiropyran chromophore. Reprinted with permission from Edahiro et al. (2005). Copyright 2005 American Chemical Society.

Light irradiation may also drive conformation changes of sensitive moieties. Displacement caused by the change in molecular conformation can be exploited to alter the chemical-physical properties of material surfaces. Spiropyran or azobenzene are two molecules that were extensively used for this specific purpose (Klajn, 2014; Fedele et al., 2018; Landry et al., 2018). In the context of cell adhesion, Higuchi et al. coated glass plates with a copolymer of nitrobenzospiropyran and methyl methacrylate (Higuchi et al., 2004). This copolymer can be subjected to the reversible transformation from a hydrophobic spiro conformation to a hydrophilic zwitterionic merocyanine isomer by means of irradiation of UV light. The authors first demonstrated the reversibility of the transition (which required at least 24 h in a dark environment) and then proved KUSA-1 cells detachment after a 4-min irradiation with light. Additionally, the authors also reported fibrinogen and platelet detachment. Edahiro et al. synthesized a photoresponsive cell culturing surface composed of poly(N-isopropylacrylamide) with nitrospiropyran as actuating element attached as side chain on the supporting polymer (Edahiro et al., 2005) (Figure 5). The authors showed that CHO-K1 cells persisted on the UV irradiated regions of the material, whereas a low temperature washing detached cells. The system proved to be reversible as irradiation with visible light at 400–440 nm released the cells immobilized on the preceding UV exposure. While this system is sufficiently versatile enabling spatial patterns of cell adhesion sites, it requires additional steps of low temperature cell washings to achieve a complete reversibility.

**Figure 6:**
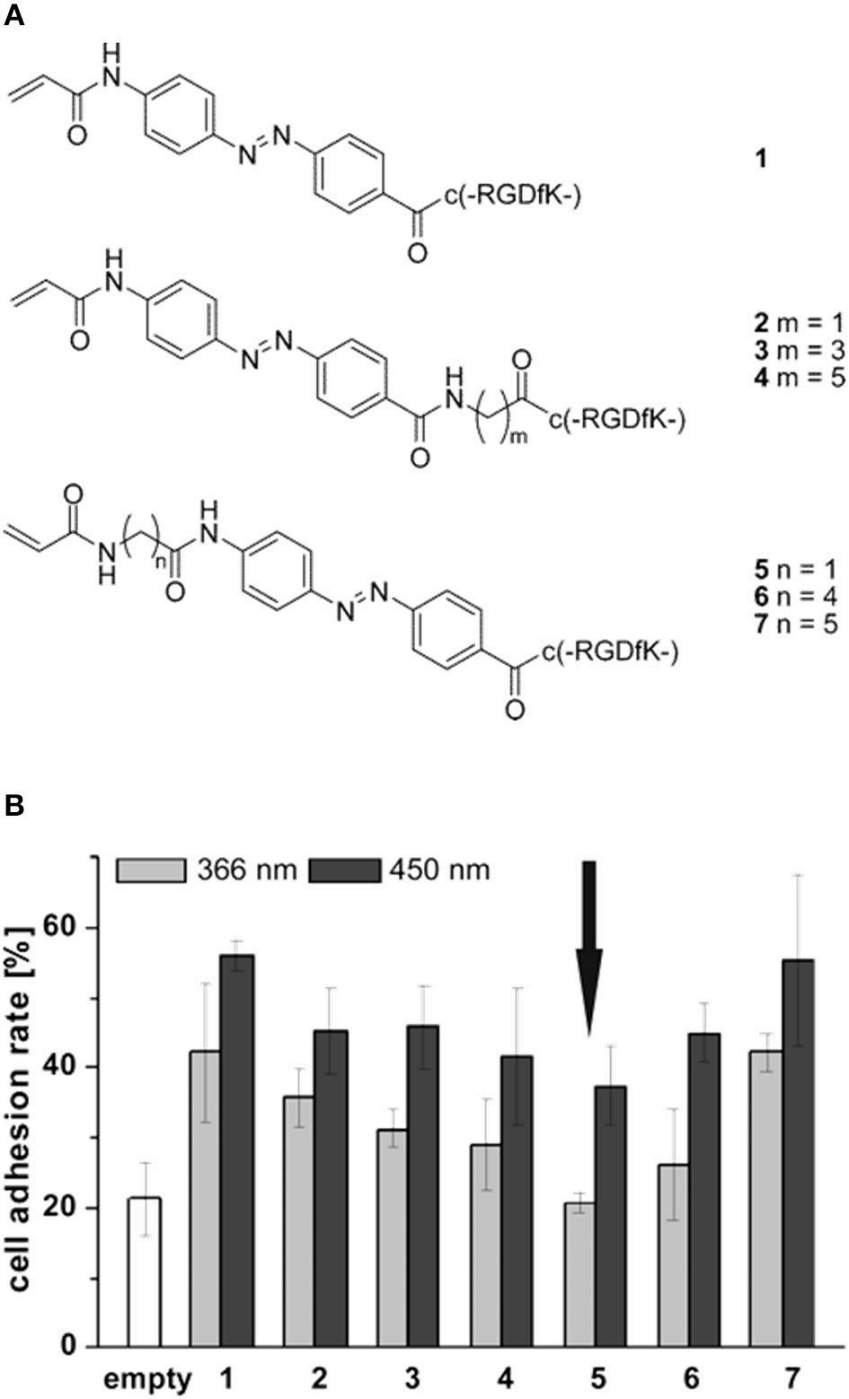
**(A)** Set of cyclic RGD peptides tethered to a photoswitchable 4-[(4-aminophenyl)azo]benzocarbonyl unit. Spacer length varies according to m or n value and on the isomerization of the switch. **(B)** Effects of spacer length and conformation on MC3T3 adhesion on PMMA. Substrates were irradiated for 3 h at 450 nm (dark gray column) and overnight at 366 nm (light gray column), respectively. Arrow indicates comparable level of cell adhesion with respect to the uncoated material (white column). Adapted with permission from Auernheimer et al. (2005). Copyright 2005 American Chemical Society.

The light induced *cis-trans* isomerization of azobenzene derivatives has been extensively used into biopolymers to change their structure in a controlled manner (Zhang et al., 2017). Exploiting this peculiar characteristic Auernheimer et al. were able to change distance and orientation of RGD ligands thus affecting cell attachment on poly(methyl methacrylate) (Auernheimer et al., 2005). The authors used 4-[(4-aminophenyl)azo]benzocarbonyl photoswitch as actuating element of spacers with different lengths containing an acrylamide anchor and a cyclic RGD tail (Figure 6A). Improved adhesion of MC3T3-E1 preosteoblasts were observed for all the spacers used when the azobenzene was in the *trans* configuration (i.e., after irradiation at 450 nm). Conversely, a general decrease in adhesion was observed when the azobenzene was in the *cis* state, which caused spacer shortening and decreased accessibility to the ligand (Figure 6B).

Similar to the approach of Auernheimer et al., Liu et al. designed and fabricated SAMs an Au containing alkanethiols with azobenzene groups and functionalized with GRGDS (Liu et al., 2009). Changing the conformation of the azocompound through light irradiation caused the ligand to be either exposed on the surface (in the *trans* configuration) or hidden in the SAM (in the *cis* configuration). The authors demonstrated that the SAM reversibly enabled/impaired adhesion of NIH3T3 in a time-frame of few hours. Also, in this case, changes in the orientation and conformation occurring at the molecular level are sufficient to alter cell adhesion. Adhering cells could be detached by supplementing the culture medium with soluble GRGDS at pH 8.0. In this condition, the soluble ligand competes with the bound one. After detachment azomolecules could be switched to the *cis* isomer thus hiding the ligand and reducing cell attachment consequently. After irradiating with visible light, azomolecules reverted to the *trans* form, which once again enabled cell adhesion. In this case reversibility is subjected to additional steps therefore this method cannot be considered as fully reversible. RGD ligand burying into a polyelectrolyte multilayer (PEM) was exploited to reversibly modulate NIH3T3 cells adhesion with UV light irradiation (Goulet-Hanssens et al., 2012). Differently from solid substrates, PEMs enable modifying the stiffness according to the processing conditions such as layer numbers and pH.

Changes in azomolecule conformation were also exploited to modulate adhesion on SAMs through a host-guest approach (Gong et al., 2011). Gong et al. synthesized α-cyclodextrins on alkanesilane molecules to form SAMs on SiO_2_. This template enabled the formation of inclusion complexes with azobenzene-GRGDS via host-guest recognition. Stable complexes were formed when the azomolecule was in the *trans* configuration allowing ligand display. In this setup HeLa cells effectively adhered and spread on the substrates. However, upon irradiation with UV at 365 nm for 10 min (a condition not harming the cells) caused an extensive cell detachment.

### Temperature Controlled Presentation of Ligands

**Figure 7:**
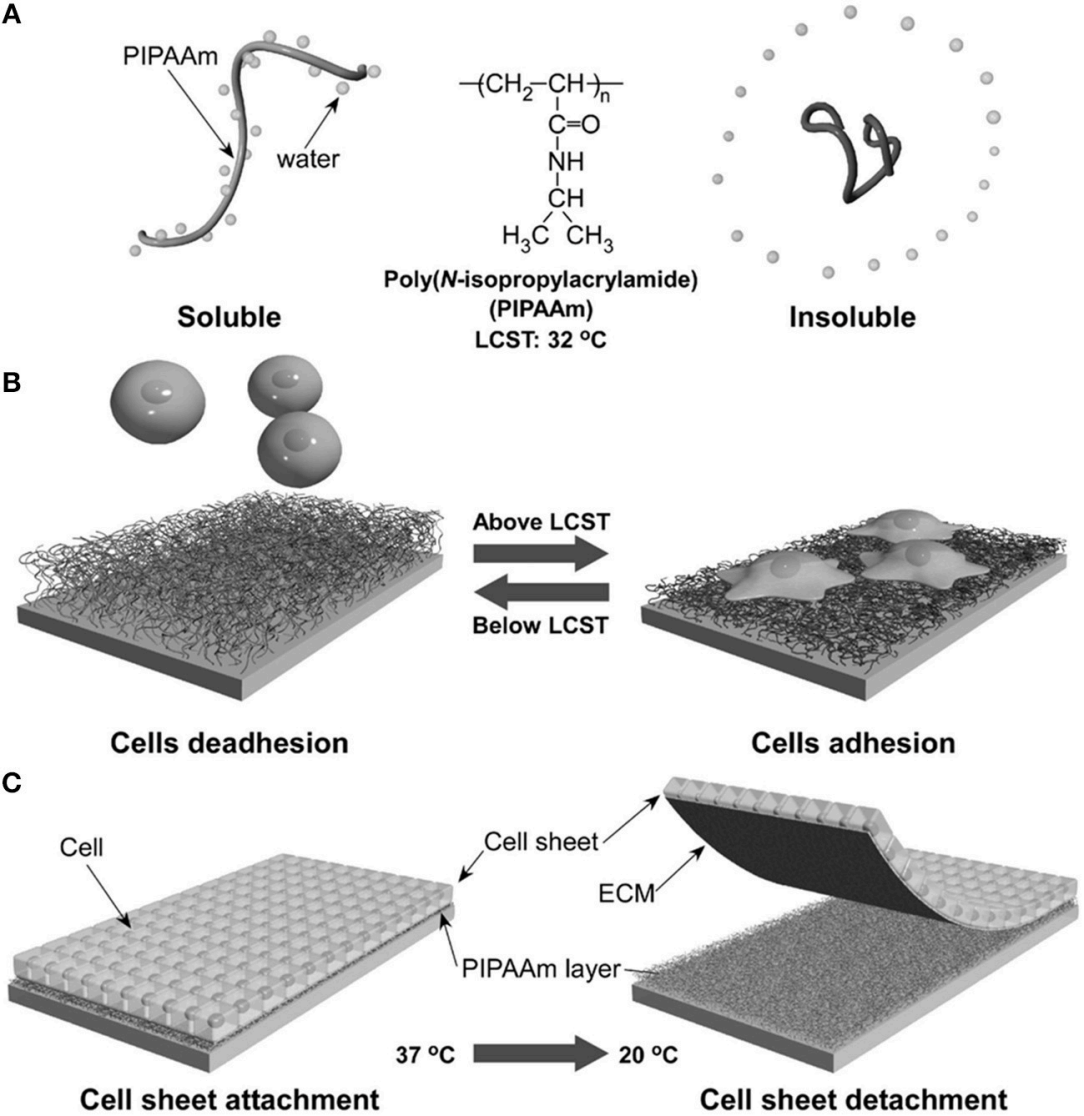
Schematic illustration of the temperature-responsive behavior of PNIPAM. In aqueous solution and above LCST, PNIPAM is stabilized by hydrophobic interactions excluding water **(A)**. This causes FN adsorption and cell adhesion. Conversely cooling below the LCST promotes water inflow and FN release with consequent cell detachment **(B)**. Confluent layers of adhering cells can detach from PNIPAM-grafted substrates when decreasing temperature from 37 to 20°C **(C)**. Reprinted from Tang and Okano (2014).

The ability of certain polymers to significantly change conformation and packing with temperature was exploited to modulate polymer-protein interactions and hence cell adhesion to the polymer layer. One of the most effective temperature responsive polymeric systems for cell cultures is the poly(N-isopropylacrylamide) (PNIPAM) showing a lower critical solution temperature (LCST) around 32°C, a temperature that does not alter cell viability significantly. Okano's group pioneered the use of PNIPAM to revert the adhesion of confluent cell layers enabling their detachment from culturing plates (Yamada et al., 1990). This represents a crucial step for harvesting cell layers for cell sheet engineering processes. Briefly, electron beam grafted PNIPAM onto conventional PS dishes allows cell adhesion and proliferation at temperatures above the LCST. In this case, PNIPAM is stabilized by hydrophobic interactions that exclude water from the polymer network (Figure 7A). Fibronectin firmly adheres to the hydrophobic network promoting cell adhesion. When cooled below the LCST, interchain H-bonds form allowing network swelling with the inflow of water. This conformational change causes the desorption of FN and subsequent detachment of the cells. The system can be reversibly made hydrophobic by re-heating the network above the LCST (Figure 7B). Such an elegant method proved to be effective in detaching large confluent cell sheets without applying mechanical stresses possibly harmful for the cells (Tang and Okano, 2014). This enables the manipulation of macroscopic layers for cell sheet engineering applications (Figure 7C). The same group demonstrated a similar mechanism of action by conjugating RGD on temperature responsive P(NIPAM-co-2-carboxyisoprpylacrylamide) (Ebara et al., 2004). Cell adhesion, spreading and proliferation were observed above the LCST. Below this threshold, however, network swelling caused mechanical disruption of RGD-receptor couplings along with shielding RGD sequences from integrin engagement. Generally, changes in temperature cannot be focused in selected part of the samples, therefore local actuation cannot be achieved straightaway. However, conjugating signal patterning and temperature responsive polymers partly solved this issue (Williams et al., 2011).

### Enzyme and Mechanical Control of Ligand Presentation

Cells contribute with external stimuli and insults to reconfigure ECM structure dynamically. Common strategies cells pursue to remodel the environment include the secretion of enzymes cleaving ECM proteins and the application of mechanical forces (Janmey and Miller, 2011; Bonnans et al., 2014). Enzymes are characterized by a high selectivity and work in mild, physiologic conditions, making them ideal candidates to trigger the activity of responsive biomaterials. Using the approach of exposing adhesion sites with proteolytic enzymes, Todd et al. exploited the cleaving activity of chymotrypsin to convert a cell repellent polyethylene glycol (PEG)-based polymer film into a bioactive support (Todd et al., 2007). Briefly, a PEG-acrylamide film was spin coated onto an epoxy-functionalized glass. RGD was attached onto the film and capped with a large 9-fluorenylmethoxycarbonylphenylalanine group. The exposure of the film to chymotrypsin caused the enzyme to selectively remove the capping group making the RGD ligand available for integrin engagement and cell adhesion. This work demonstrated the feasibility of exploiting the biological activity of molecules to activate dynamic platforms, paving the way toward the development of systems activated on-demand by cell secreted molecules. However, while this system can be conveniently classified as dynamic, it is non-reversible since once unprotected by the enzyme activity the ligands cannot be capped reversibly.

More recently, Roberts et al. exploited enzyme switchable substrates to investigate and manage the delicate adhesion/cytoskeleton balance regulating quiescence or differentiation of human multipotent mesenchymal stromal cells (hMSCs) (Roberts et al., 2016). Glass substrates were functionalized with multifunctional chains constituted by a PEG linker, an RGD peptide, a dialanine cleavable site and either a fluorenylmethyloxycarbonyl blocking group or a PEG adhesion reducing moiety at the distal end. The introduction of elastase cleaves the dialanine site exposing the RGD that allows the transition from a “low” to a “high” adhesion state. hMSCs on platforms in a “low” configuration express β_1_ rich FAs and negligible bone morphogenetic protein (BMP) receptor colocalization. Conversely, hMSCs on enzyme activated substrates expressed β_5_ rich FAs and increased BMP signaling. Altogether, these data suggest that low (but not null) adhesion prevents metabolome activation for osteogenesis, whereas high adhesion increases BMP signaling through increased levels of intracellular tension. This work demonstrated how delicate the adhesion/tension balance is and subtle perturbations can result in differentiation of stemness maintenance. Only advanced dynamic platforms as the one here described can provide a reliable testing platform to unravel the complex mechanisms underpinning fate decisions.

Several natural biomolecules change their affinity with other molecules or functions when subjected to mechanical stretching (Sawada et al., 2006; del Rio et al., 2009; Singh et al., 2010; Janoštiak et al., 2014). Exploiting such a biomimetic route Bacharouche et al. fabricated an elastomer-based device in polydimethylsiloxane (PDMS) whose adhesion properties changed dynamically and reversibly according to the device deformation (Bacharouche et al., 2013). The authors grafted in sequence PEG chains under elastomer stretching and then RGD was conjugated to chains. In a relaxed state the ligand resulted embedded in the PEG brushes. When the elastomer was subjected to stretching, RGD became exposed and accessible. Human osteoprogenitor cells firmly adhered to the PDMS in the stretched form and expressed talin rich and well-defined FAs. In the relaxed state cells became round showing a diffuse talin staining all along the cortical region. This suggests that integrin disengagement from the concealing ligands destabilize FAs thus leading to cell detachment. Surface deformation is also responsible for the redistribution of material elements. For example, uniaxial deformation causes material elongation along the stretching direction and a contraction in the orthogonal direction. Following this observation Deng et al. investigated how cells reacted to continuously and reversibly variable interligand spacings (Deng et al., 2017). They transferred a quasi-hexagonal pattern of RGD functionalized gold nanoparticles spaced 35 nm apart (a spacing permissive for adhesion formation) on a highly stretchable poly(N-acryloyl glycinamide) hydrogel. Macroscopic stretching causing a ligand separation above 70 nm impaired the formation of stable adhesions in MC3T3 cells even if the spacing in the orthogonal direction was below 70 nm. Reversing stretching-relaxation forced the REF 72 cells to first acquire a polarized shape and a highly motile behavior and then (after relaxation) to display a spread shape and more stationary behavior. Therefore, not only cells show an extraordinary sensitivity to nanoscale spacing of ligands, but also they finely discriminate spatial variations in the distribution.

### Cell-Mediated Remodeling of Ligand

The platforms illustrated so far enable a dynamic display of signals, but do not allow a cell mediated, spatio-temporal redistribution of these. This becomes a critical issue for investigating biologic phenomena such as cell remodeling of ECM or for assessing the role of receptor clustering in signal transduction. To address these issues, platforms based on lipid mono or bilayers proved to be particularly effective. While these may represent the ideal systems to study the dynamics of cell-cell adhesions, various chemical strategies have been developed to functionalize lipidic membranes with matricellular cues (Gooding et al., 2014; Koçer and Jonkheijm, 2018). Molecules incorporated in the layer are endowed with a lateral mobility, whose dynamics is affected by the chemical composition of the layer. Concerning lipid bilayers, those usually employed as culturing substrates are made of 1,2-dicoleoyl-sn-glycero-3-phosphocholine (DOPC) that possess a low gel-fluid transition temperature, indicating a higher lateral molecular diffusion. Conversely, membranes of 1,2-dipalmitoyl-sn-glycero-3-phosphocholine (DPPC) are more thermally stable with less diffusible molecules (Lindblom et al., 2006). Culturing substrates based on bilayers are conveniently formed on hydrophilic surfaces (supported lipid bilayers, SLBs). SLBs provide a good level of spatial control of ligand positioning when physical constraints are introduced on the nanostructured support on which the membranes are formed (Groves, 1997). In this case, nanometric relieves of the substrate locally impair lateral mobility of molecules, thus enabling clustering. Following this approach Yu et al. investigated the early adhesion events and lateral clustering of integrins (Yu et al., 2011). RGD functionalized DOPC membranes were formed on a glass substrate containing 5 × 100 nm metal wires spaced by 0.5–4 μm gaps. Thanks to lateral diffusion and capture provided by the nano-barriers, the authors observed integrin recruitment and the formation of submicron clusters in which adhesion proteins such as talin, paxillin, and FAK were formed in a contraction-independent manner. This preceded actin filament assembly at cluster sites, for which, myosin contraction produced even longer clusters. Unraveling such a complex dynamics required the versatility and fluidity of SLB as opposed to conventional ligand-immobilized substrates. Along this line, Vafaei et al. have recently compared the biological activity of FN and Collagen type I covalently anchored to DOPC SLBs with that of proteins non-specifically adsorbed onto SiO_2_ substrates (Vafaei et al., 2017). The authors found an increased flexibility of the proteins and more efficient cell adhesion and proliferation, providing further evidence that SLBs may represent a more biomimetic microenvironment to study certain biological processes, respect to synthetic platforms not allowing ligand displacement.

SLBs have also been employed to address the role of ligand clustering on stem cell fate decision. Koçer and Jonkhejim correlated hMSCs spreading with receptor clustering and cell differentiation (Koçer and Jonkheijm, 2017). To modulate lateral mobility either DOPC or DPPC membranes functionalized with RGD were used. hMSCs showed higher adhesion, higher level of expression of osteogenic differentiation markers and calcium deposits on DOPC membranes suggesting a positive role of ligand clustering and integrin activation in dictating stem cell fate decisions. Additionally, SLBs proved to be very stable as receptor recruitment and differentiation occurred on very different timescales (minutes vs. days) and yet they allowed cell culture for up to 10 days.

## Dynamic Topographies

Static topographic relieves in the form of micro/nano pits, protrusions or channels dramatically affect various aspects of cell behavior including, migration, proliferation and differentiation (Dalby et al., 2014; Ventre and Netti, 2016b). Several technologies have been developed to perform systematic studies of response to topographic signals of a sufficiently large number of cells. Among these, replica molding, nanoimprint lithography, block copolymer micelle nanolithography exhibit a reasonable balance between features fidelity and resolution and large area patterning (Ventre et al., 2018). Extending the studies in a dynamic framework requires the development of stimuli responsive materials, along with manipulating/actuating strategies that must be both effective and cell compatible. The examples of surfaces displaying adhesion signals dynamically all share the common trait that the switches act on a molecular level (capping, conformation changes, desorption). For the successful implementation of dynamic topographies, i.e., relieves changing shape and height, the coordinated motion or matter removal on a submicro- or micro-scale level is necessary. Specific chemical strategies need to be developed for allowing this type of material transformation as well as the triggering stimulus must be effective on a reasonable time scale.

### Mechanical Control of Topographic Patterns

An early example of dynamically changing topographic pattern was proposed by Zhu et al. who fabricated a thin silica-like layer on a PDMS elastomer with radiofrequency oxygen plasma (Zhu et al., 2005). The silica layer was rendered protein repellent via chemical vapor deposition of (tridecafluoro-1,1,2,2-tetrahydrooctyl)-1-trichlorosilane and incubation with Pluronic F108. Cyclic stretching of the elastomer caused the thin brittle layer to form parallel arrays of cracks (having width ranging in the 0.1–3 μm interval) on which C2C12 myoblasts adhered and elongated. Cells subjected to cyclic opening-closing of the cracks responded with sequential changes in elongation/retraction. A similar approach was reported by Lam et al. who fabricated patterns of periodic microscale waves by compression-induced buckling of a brittle thin film on the surface of a PDMS elastomeric substrate (Lam et al., 2008). C2C12 cells adhered and retraced the contours of the wavy pattern when the elastomer was compressed, whereas cells displayed a random orientation when the PDMS strain was released. These are elegant examples illustrating the fabrication of dynamic substrates in a simple and cost-effective manner. However, with these strategies it is not possible to achieve a fine control on the features of the topographic patterns. Also, the geometry of the pattern is limited to straight channels and cannot be changed readily.

Guvendiren and Burdick further elaborated this concept by developing a method to spatially control the geometrical features of the strain responsive topographies (Guvendiren and Burdick, 2013b). Exposing to ultraviolet/ozone uniaxially or biaxially stretched PDMS sheets resulting in the formation of a stiffer outer skin. Selective release of the strain produced a wave pattern perpendicular to the strain direction. Release of both directions of strains in the biaxially stretched sheets resulted in a labyrinthine pattern. Also, local masking irradiation enabled site-specific patterning. hMSCs cultivated on the dynamic patterns responded to the switching by altering shape, orientation and nuclear area. Although very effective in the short culturing periods, the effectiveness of the dynamic topographies decreased in time as cell proliferation and cell-cell contacts overruled the topographic guidance.

### Light Responsive Topographies

Material degradation induced by light irradiation enables embossing topographic structures with a higher spatial control of the features with respect to the methods described above. Kirschner and Anseth synthesized a photodegradable PEG based hydrogel by coupling nitrobenzyl-based photodegradable acrylate to PEG-bis-amine and PEG macromer (Kirschner and Anseth, 2013). Irradiating the gel with UV light at 365 nm caused photolabile bond breaking and gel erosion. Using photomasks, the authors were able to direct erosion giving rise to topographic patterns whose depth was proportional to the irradiation time. Furthermore, by directing the laser beam of a multiphoton microscope multiple patterns were embossed on the same gel. Dynamic patterning in presence of cells was demonstrated by cultivating hMSCs on the top of a FN coated photolabile hydrogel. Microscale channels were imprinted on the gel which caused the cells to reorient, eventually increasing their aspect ratio. Afterwards, a grating of regular squares was produced by drawing lines orthogonal to the primary pattern. This restored a symmetric condition that induced cells to acquire a round shape. This platform proved to be highly versatile as it enabled writing multiple patterns with different features on the same substrate. Additionally, the patterning procedure along with the hydrogel degradation products does not harm cell. However, once sculpted hydrogel surfaces cannot acquire the initial shape; this may pose severe limitation on the reversibility of the system.

Thanks to their ability to change conformation upon light irradiation, azopolymers have been used in various biological applications (Beharry and Woolley, 2011; Wei et al., 2015; Goulet-Hanssens et al., 2016). One of the first examples of using azopolymers as cell culturing substrates was provided by Baac et al. (2004). Laser holography was used to imprint undulated nanoscale patterns on the commercially available light responsive azopolymer poly[(methylmethacrylate)-co-(Disperse Red 1 acrylate)]. Azopolymeric patterned films proved to be biocompatible enabling the attachment of different cell types. This paved the way to use patterned light to modify surface topography of films dynamically. An early attempt to inscribe a topographic pattern on an azopolymeric cell culturing substrate while preserving cell viability was reported by Barillé et al. (2011). The authors reported pattern inscription via irradiation with a two-laser beam interferometric pattern or through molecular self-organization induced by a single beam. PC12 cells reacted to the flat-grooved transition by elongating and aligning along the pattern direction and extending neurites. Although preserving cell viability, an influence of the liquid medium on the process of pattern inscription was reported. The issue of azopolymer stability in biologic media was addressed by Rocha et al. who noticed material reorganization in aqueous environments that varied according to the polarity and stiffness of the azopolymer (Rocha et al., 2014).

**Figure 8:**
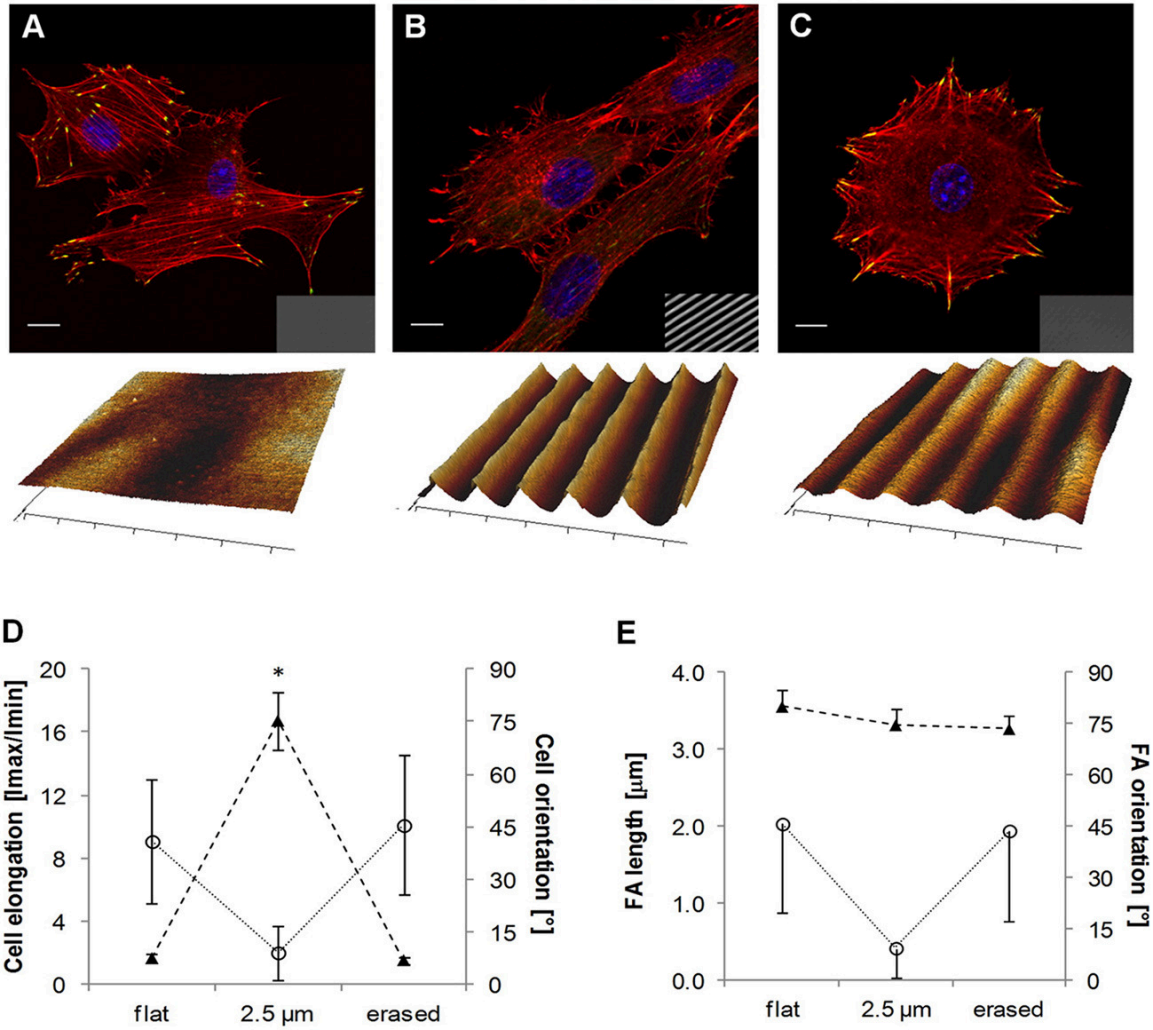
Confocal images of NIH3T3 cells cultivated on **(A)** flat pDR1m substrate, **(B)** SRG linear grating, and **(C)** pattern-erased substrate with circularly polarized light. Insets in the bottom right show images in transmission. Representative AFM scans are reported below each image. **(D)** Plot of cell elongation (solid triangles) and cell orientation (open circles). **(E)** Plot of FA length (solid triangles) and orientation (open circles). The asterisk indicates a significant difference with respect to the flat case. Bars indicate the standard error of the mean in the case of cell elongation and FA length, whereas they represent the standard deviation in the case of cell and FA orientation. Reprinted with permission from Rianna et al. (2015). Copyright 2015 American Chemical Society.

**Figure 9:**
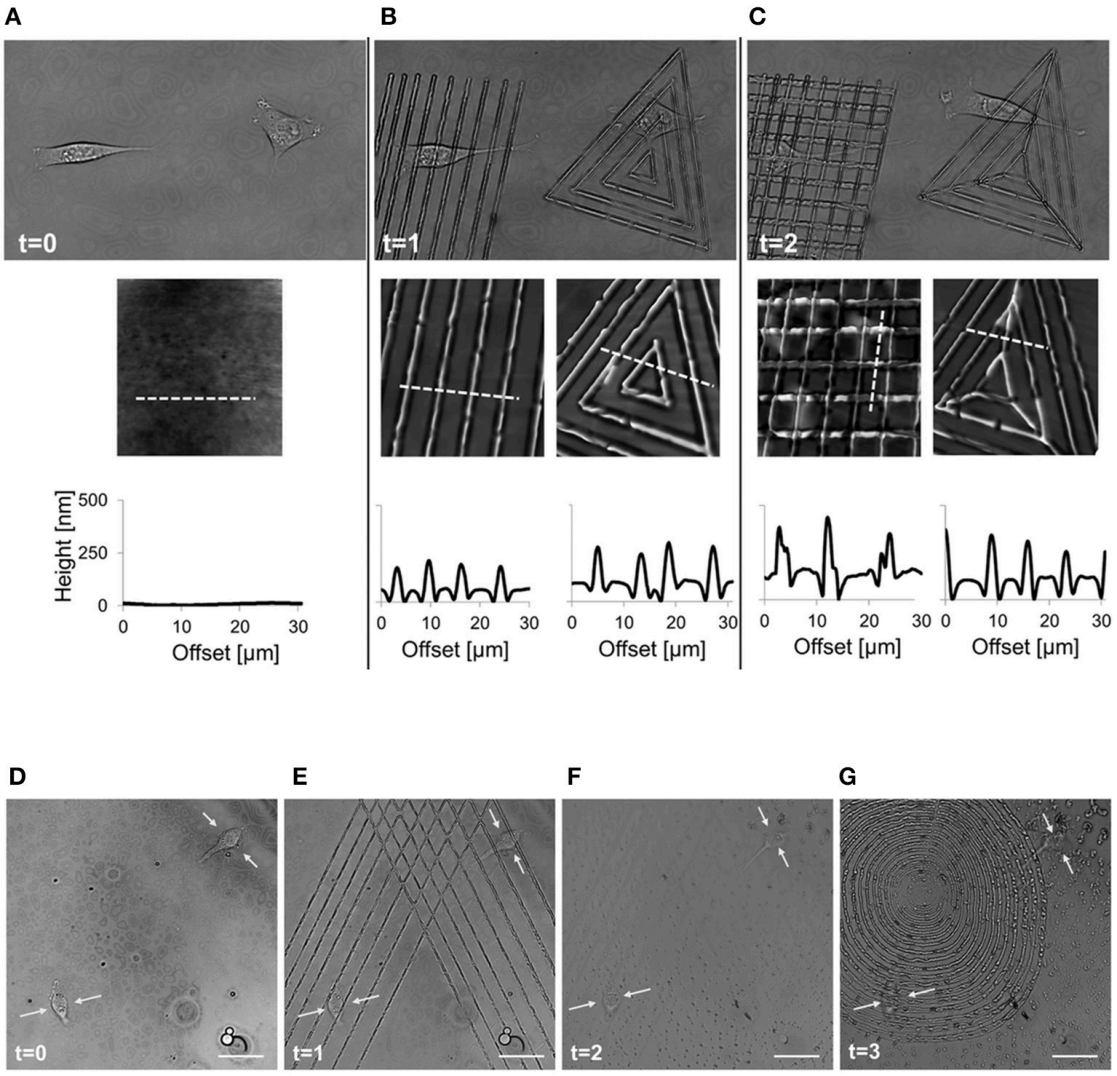
Spatio-temporal control of pattern inscription while preserving NIH3T3 viability. **(A)** Transmission images of two non-interacting cells on flat pDR1m substrate (*t* = 0); **(B)** two different topographic patterns were inscribed on the same location by means of a 514 nm laser, with a 30 s inscription time (*t* = 1); **(C)** after 45 min a second topographic pattern was superimposed to the primary one (*t* = 2). On the bottom of each image, a representative AFM scan and related cross-section profiles are reported. Demonstration of the feasibility of the pattern inscription-erasure process while preserving cell viability. **(D)** Confocal images of two cells non-interacting cells on flat pDR1m substrate; **(E)** Primary pattern inscription with 514 nm laser 30 s inscription time; **(F)** pattern erasure by employing unpolarized and incoherent light for 60 s after removing the medium; **(G)** second pattern inscription with 514 nm laser 30 s inscription time. Images were collected at time zero (before patterning) and later at three different time points. Yellow arrows point to the central portion of the cell body during the entire process. Scale bars are 20 μm. Adapted from Rianna et al. (2016). Copyright Wiley-VCH Verlag GmbH and Co. KGaA. Reproduced with permission.

More recently Rianna et al. investigated the feasibility of employing Poly(Disperse Red 1 methacrylate) (pDR1m) as a suitable substrate to emboss topographic patterns reversibly for cell culture experiments (Rianna et al., 2015). Preliminary tests to assess pattern stability under conditions comparable to those experienced during cell culture, were performed. Patterns in the form of microgrooves or microgrids were inscribed on films by using an interference pattern of light. Cells were mostly round when cultivated on flat or grid patterns, whereas they appeared to be highly elongated and aligned along the direction of linear microgrooved patterns (Figures 8A,B,D). Circularly polarized light was used to induce pattern erasure on pDR1 films, which caused a sharp decrease in the elongation of cells once cultivated on the erased pattern (Figures 8C,D). FA length did not display changes in the writing/erasing cycles, whereas FA orientation was very sensitive to the topography as parallel FAs were observed on the SRG only (Figure 8E). Authors from the same group further elaborated this concept by integrating pDR1 patterned with a single photon laser with the aim of developing dynamic substrates to study how spatio-temporal variations of topographic patterns affect the behavior of vital cells in real-time (Rianna et al., 2016). A conventional confocal microscope was used to guide laser beam path to emboss complex patterns on pDR1m thin films with submicrometric resolution. Fibroblasts cells responded to the dynamic patterns by altering their morphology and migration because of cytoskeleton and focal adhesion reorganization. Furthermore, irradiating the substrate with an incoherent and unpolarized light of a mercury lamp enabled the erasure of topographic patterns on cell-populated pDR1m. A further peculiarity of the confocal technique consists in enabling multiple pattern inscriptions on the same platform simultaneously, so that isolated cells can be exposed to different patterns in real-time (Figures 9A–C). The possibility to imprint and erase several topographic patterns on azopolymer films with an easy and versatile method may pave the way to an investigation of complex processes involved in cell material-crosstalk (Figures 9D–G). Rossano et al. have recently exploited this technique to study NIH3T3 fibroblast response to dynamic circular topographic patterns (Rossano et al., 2018). The authors found that cells reacted to transition from flat to circular topographies quickly and that patterns of circular ridges 2 μm wide and spaced of 10 μm most effectively affected cell shape and local orientation. Since stress fibers and FAs cannot grow in a bent fashion, circular topographies proved very effective in destabilizing cytoskeletal structures and hence in decreasing the mechanical properties of cells.

Koçer et al. integrated microscale topographic cues with dynamic and reversible surface nanoroughness by exploiting the light induced azobenzene isomerization within liquid crystal networks (Koçer et al., 2017). More specifically, the polymer network containing the azobenzene moiety was photocrosslinked in a network whose microstructure was in the cholesteric phase. UV irradiation decreased the order of the phase causing an increase in surface nanoroughness, from 9.0 ± 1.2 to 11.1 ± 1.2 nm. NIH3T3 cells cultivated on the dynamic surfaces dynamically changed the migration behavior from motile to stationary according to the underlying topography.

### Temperature Responsive Topographies

Biocompatible shape memory polymers (SMPs) enabled to investigate cell recognition and response to time changing topographies by exploiting consolidated and inexpensive technologies such as casting and hot embossing (Ratna and Karger-Kocsis, 2008; Meier et al., 2015). Generally, SMPs can recover permanent shapes upon stimulation of temporary ones. The latter can be implemented by means of mechanical deformation, whereas the application of an external stimulus, including heat, light, or solvent exposure promotes the recovery of the permanent shape (Mather et al., 2009). SMPs possess the advantage of retaining microscale topographic features, with high fidelity and—if conveniently engineered—SMPs can switch under cytocompatible conditions (Le et al., 2011). One of these materials is polycaprolactone (PCL) that is able to change topography if heated above the transition temperature (*T*_*trans*_) of 40°C (Le et al., 2011; Ebara et al., 2012). In one example, Le et al. crosslinked star shaped PCL liquid prepolymer under UV light in presence of diethoxyacetophenone photoinitiator into specifically designed molds, thus allowing the material to acquire the equilibrium primary topography. Mechanical forces were applied above *T*_*trans*_ to impress the transient, secondary topography, and cooled down the *T*_*trans*_. Reduction of the molecular mobility prevented the material to relieve the mechanical stress, thus enabling the secondary topography to remain impressed. Heating above *T*_*trans*_ provided sufficient mobility to the polymers to restore the original shape. The authors exploited this to investigate the response of hMSCs to dynamically changing microtopographies. Cells seeded on temporary 3:5 μm grooved pattern elongated and aligned along the pattern direction. However, when the substrate was heated above *T*_*trans*_ the primary-planar shape emerged, causing the cell to acquire a stellate morphology. While this method is simple (not requiring expensive device to actuate the transition) and effective, it is not truly reversible as secondary shapes cannot be reacquired. Furthermore, cells on secondary topographies need to be cultivated below the *T*_*trans*_ (28°C) which might be an issue for long-term cultures. Beside adhesion and orientation, patterned SMPs were shown to dynamically regulate the structural and contractile properties of cardiomyocytes (Mengsteab et al., 2016). Neonatal rat ventricular myocytes (NRVMs) were cultivated on a PCL-based SMP exhibiting a primary nanogrooved pattern (400 nm wide ridges; 500 nm grooves; 150 nm depth) and a secondary transient nanogrooved pattern with same dimensions but rotated of 90° with respect to the primary one. This design was aimed at recapitulating the orientation of collagen fibrils in the myocardium. NRVMs on the transient nanopattern exhibited a homogeneous population of elongated FAs, nuclei alignment along the pattern direction and a unidirectional and a homogeneous contractile behavior. The surfacing of the primary pattern determined an increased dispersion of FA orientations and after 48 h cells showed a bimodal distribution of the directions of contraction. These data suggest that nanotopographic patterns do not dictate organization and contractile properties permanently making dynamic topographies a suitable signal to alter the organization of cells on a collective level.

Another class of polymers exhibiting shape memory are the crosslinked polyurethane-based adhesives, commercially available under the name NOA63. Davis et al. processed NOA63-based films displaying dynamic topographies. A flat surface was recovered from a microgrooved pattern by changing the temperature from 30 to 37°C (Davis et al., 2011). Such a temperature preserved cell viability. Mouse embryonic fibroblasts accompanied the transition by remodeling their cytoskeleton and changing their shape accordingly. A drawback of this method relies in the long time-frame required for the topographic switch to occur (~hours).

## Dynamic Stiffness

Materials whose mechanical properties can be changed dynamically represent unique platforms to investigate complex biological phenomena such as morphogenesis and wound healing. In fact, the mechanical properties of the culturing microenvironment—and more specifically its stiffness—are known to potently regulate cell differentiation (Engler et al., 2006; Ivanovska et al., 2015). However, how changes in ECM mechanics accompany or anticipate physiological or pathological states, are not thoroughly understood and artificial platforms recapitulating the dynamics of stiffness changes *in vitro* would be of great importance for studying the development of physio-pathological states. ECM stiffening is a typical phenomenon accompanying several biologic processes such as *in vivo* organogenesis or the progression of pathologies (Berry et al., 2006; Georges et al., 2007; Krieg et al., 2008). Dynamic changes of substrate stiffness usually rely on degradation or crosslinking of hydrogels that can either proceed spontaneously or can be activated by external triggers such as light.

By exploiting photodegradation, Kloxin et al. aimed at addressing myofibroblastic differentiation in response to microenvironment softening (Kloxin et al., 2010). Fibroblast to myofibroblast differentiation is a crucial step in wound healing. Wounds are highly dynamic environments (in terms of biochemical microenvironment, architecture and mechanical properties) and today the role of biochemical/biophysical signals in governing the dynamics of healing is poorly understood. Dynamic platforms may provide valuable insights into the biology of regenerative/reparative processes. To this aim, valvular interstitial cells were cultivated on a PEG based hydrogel containing photodegradable crosslinkers. The photodegradable hydrogel allowed to change moduli in the physio-pathologic range within minutes after irradiation with UV light. Cells cultivated for 5 days on stiff (32 kPa) hydrogels displayed the characteristic phenotype of activated myofibroblasts, whereas cells cultivated on soft (7 kPa) gels were in quiescent form. However, the stiff to soft transition (3 days on stiff, followed by 2 days on soft) was sufficient to deactivate cells.

Not only the absolute magnitude of the stiffness affects fate decisions, but the way the mechanical stimulus is presented also plays an important role in regulating the differentiation process. Along these lines, Yang et al. used a photodegradable polyethylene glycol di-photodegradable acrylate hydrogel functionalized with RGD to investigate whether stem cells possess a “mechanical memory”, i.e., stem cells make fate decisions based on the temporal integration of the perceived dynamic mechanical stimuli (Yang et al., 2014). To this aim, hMSCs were subjected to a mechanical dosing, i.e., they were cultivated on stiff substrates for different time intervals (0, 1, 5, or 10 days) and then the substrate was softened down to 2 kPa. hMSCs supplemented with a small dose of mechanical stimulus (1 day) exhibited transient levels of genes involved in osteogenesis. Conversely, an irreversible activation of the differentiation programme was observed when cells were cultivated for 10 days on stiff hydrogels prior to softening. The authors suggested that stem cells do possess a mechanical memory for which Yes-associated proteins/Transcriptional coactivators with PDZ-binding motif (YAP/TAZ) acts as mechanical rheostats since they persist in the nucleus, hence promoting osteogenesis, if cells are continually exposed to stiff environments.

In the context of addressing the role of dynamic changes of material stiffness on stem cell differentiation, Guvendiren and Burdick developed a methacrylated hyaluronic acid (HA)-based gel whose stiffness can be increased through light-mediated radical polymerization (Guvendiren and Burdick, 2012). hMSCs were cultivated on soft gels for selected time intervals, after which gels were stiffened by light irradiation. Cells quickly reacted to material stiffening by increasing area and the expression of contractile forces. Furthermore, early stiffening (i.e., cells exposed to a stiff environment for a long timeframe) promoted osteogenesis, whereas late stiffening promoted adipogenesis. Besides differentiation, the timing of the materials stiffening proved to be a crucial parameter for activating pathological states in *in vitro* models (Caliari et al., 2016). Using hepatic stellate cells cultivated on a methacrylated HA gel sensitive to blue light irradiation (~470 nm), Caliari et al. showed that a soft-to-stiff transition occurring after 6 days prompted an accelerated signaling kinetics of both YAP/TAZ and alpha-smooth muscle actin whose time-course was similar to observed *in vivo* activation dynamics. Anticipating the transition at day 1 did not produce the same trend.

These examples suggest that the parameter stiffness has not to be intended as a “state variable” for which the cell response only depends on the initial and final values of the stiffness, being independent from the specific pathway that led to the change. The systems described above operate on step-wise changes of the stiffness occurring in short-timeframes (minutes-hours). Cells are exposed to continuous changes of the *in vivo* biophysical microenvironment (Montell, 2008; Bonnans et al., 2014). Therefore, it is likely that the kinetics of stiffness variations may also play a role in affecting cell functions and fates. To address this, it is necessary to develop chemical modifications producing crosslinks or bond breakings whose reaction kinetic eventually match the time-variations observed *in vivo*. To verify whether time-dependent stiffening plays a role in establishing an adequate microenvironment for myocardial maturation Young and Engler synthesized a thiolated-hyaluronic acid hydrogel crosslinked with (ethylene glycol)diacrylate whose crosslinking kinetics and hence stiffening dynamics was tuned to match the temporal variations of stiffness observed in developing embryos (Young and Engler, 2011). Precardiac cells cultivated on collagen coated dynamic thiolated-HA gels showed significantly higher expression of mature cardiac markers and improved assembly of contractile fibers with respect to what observed when cells were plated on conventional and static polyacrylamide gels. Even though this experimental model does not allow to change mechanical properties at will, it clearly demonstrates how undifferentiated cells are sensitive to biophysical changes of the microenvironment.

More recently, hydrogels with reversible mechanical properties were fabricated through modular modifications of HA (Rosales et al., 2017). Three different moieties were used: o-nitrobenzyl acrylates (photodegradable), methacrylates (crosslinks with a photoinitiator) and RGD. Hydrogels could be softened and then hardened (or vice versa) through sequential irradiation with specific wavelengths. MSC cultivated on the HA modified gels showed a decrease in the cell area and nuclear localization of YAP/TAZ following *in situ* softening (from 14 to 3.5 kPa), whereas subsequent stiffening (from 3.5 to 28 kPa) increased the cell area and nuclear localization of YAP/TAZ. Authors of the same groups further elaborated the design of the photoresponsive system and successfully produced a supramolecular hydrogel constituted by hyaluronic acid functionalized with the photoresponsive guest-host pair azobenzene and β-cyclodextrin (Rosales et al., 2018). Azobenzene groups in the *trans* configuration form links with cyclodextrins that can be disrupted upon UV irradiation that promotes the *cis* conversion under cytocompatible conditions. Such a design possesses the advantage of decoupling changes in network mechanical properties from changes in chemical composition. This endows the system with well-defined physical/chemical characteristics suitable for studies in mechanobiology.

## Conclusions and Future Perspectives

Adhesion mediated signaling regulates various aspects of cell behavior including proliferation, migration, and differentiation. Cell adhesions have a direct impact on cytoskeleton arrangement, cell contractility, and nucleus shape, which altogether affect gene expression and eventually cell functions (Dalby et al., 2014; Murphy et al., 2014; Anderson et al., 2016; Ventre et al., 2018). Therefore, adhesion—cytoskeleton—contractility constitute a tripartite module in which changes in one element invariably induce alterations in the other thus affecting cell behavior. In this context material signals regulating cell adhesion acquire a central role, perhaps as important as chemical supplements or drugs. Culturing substrates, scaffolds or gels proved remarkable effectiveness in finely modulating even complex biological events. Yet, these culturing systems are mostly known for displaying signal in a static manner. Static signal platforms have provided tremendous insights in understanding the optimal configurations of signals to be displayed to elicit a specific response. However, in the way they are conceived static platforms cannot be employed to address other relevant questions. As soluble biochemical signals elicit very different cellular responses according to their dose and on the way they are delivered in time, do biophysical signals behave similarly? How does the variable spatio-temporal presentation of biophysical signals affect cell behavior? Does an optimal spatio-temporal presentation of bound signal to elicit a specific cell response in the most effective manner exist? Answering these questions may not only provide valuable insights for developing more effective and versatile culturing systems but might aid in shedding light on intricate biological processes involved whenever modifications in the microenvironment occur, for example in tissue and organ morphogenesis or in the development or progress of diseases. Dynamic platforms may certainly contribute to address these issues. However, many technical hurdles need to be overcome. First, a fine modulation of adhesion events and exerting a control on the dynamics of specific arrangements of FAs is necessary. Such a spatio-temporal resolution in the actuating elements of dynamic platforms has not been achieved yet. Second, dynamic surfaces have reached a level of performances that enables controlling adhesion processes at a subcellular level in time-scales that are compatible to conventional cell cultures. However, different biological responses may occur on different time scales, i.e., adhesion takes minutes to hours, whereas differentiation occurs in days or weeks. Achieving a proper time control is still an issue. Some triggers are faster than others, but once they are turned “on” or “off” the whole system responds with its own kinetics. Therefore, tuning the dynamics of the signal change to that of a specific biological phenomenon may require a careful design of the molecular switch or the integration of various switches. Third, while 2D surfaces are valuable to understand the behavior of epithelia and endothelia as they more closely mimic the native microenvironment, certain biological events specifically occur in a 3D context. Patterning signals in space with a submicron resolution is technically challenging and requires expensive equipment. Actuating switchable signals in 3D is even more complex. Laser two photon or holographic techniques might be valuable instruments for achieving a spatial control of the chemical/physical properties of the 3D environment with an adequate resolution (Applegate et al., 2015; Tong et al., 2016). Notable examples of dynamic 3D environments able to regulate cell functions and fate decisions have been already reported (Khetan et al., 2013; Das et al., 2016; Brown et al., 2018). However, integrating diverse stimuli in 3D and achieving a control on individual adhesion points still require further developments. Moreover, activating and deactivating signal exposure in a completely reversible manner is key central. Non-reversible or partially reversible systems might only partly recapitulate the dynamic behavior of the ECM or might not be entirely versatile. Creating complex spatio-temporal patterns of signals requires implementing signal display/conceal in a fast and effective manner. The uncontrolled presence of signal remnants may interfere with the intended signal presentation providing inconsistent results.

Literature data has provided compelling evidence that cell functions and fate are not dictated by a single signal type, but it is rather a complex interplay of multiple signals acting on different length- and time-scales that determines the final cell state (Dalby et al., 2014; Murphy et al., 2014). To actuate different signal acting at different timepoints, specific switches must be engineered. Photoswitches might be particularly suitable for this purpose. In fact, several molecules have been developed, for example the azoderivatives, whose response may fall within a broad range of wavelengths. In principle, by combining different light sensitive switches reacting to different wavelengths it would be possible to fabricate arrays of signals whose display could be controlled in an orthogonal manner, thus increasing the complexity of the system. This, however, requires a sapient positioning of light sensitive elements with non-overlapping absorption spectra.

Furthermore, dynamic platforms are usually engineered and fabricated to display/conceal binding sites. However, dynamic changes of the ECM are not limited to adhesion cues and involve multiple signal changes according to different chronoprogrammes. In fact, the literature on dynamic platform mostly concerns platforms regulating adhesion/detachment and migration. Yet, recent examples demonstrated enormous potentialities of dynamic signal display, particularly in what concerns fate decisions and in the acquisition of specific tissue functions (Young and Engler, 2011; Yang et al., 2014; Roberts et al., 2016). Therefore, beside exploiting soluble signals, engineering complex patterns of bioadhesive signals, the introduction of the parameter “time” acquires a crucial importance in the design of novel and more efficient culturing systems. However, dynamic surfaces, although introducing space, and time variations of biologic properties fall short of capturing more complex features of the native ECM. An intriguing strategy to tackle this issue has been recently proposed by Hay et al. who introduced the concept of bacteria-based materials (Hay et al., 2018). These finely altered the adhesive microenvironment upon external commands, eventually dictating stem cell fate decision. Non-pathogenic bacteria can be engineered as micromachines able to assemble animal proteins or release factors upon external stimulation. Nowadays, biologic genetic circuits can be integrated in bacteria to control the yield and production rate (Bittihn et al., 2018). We expect future research to be focused on integrating different strategies and technologies for the fabrication of multi-functional and dynamic platforms able to recapitulate intricate biochemical, biophysical and cellular processes that are fundamental to promote and guide biologically relevant phenomena. Applications like organoid generation or the development of pathological models would largely benefit from these advancements. Therefore, we expect that the field of dynamic platform engineering can potentially contribute not only to unravel complex biological events such as migration and differentiation, but could impact the medical and pharmaceutical areas thanks to the development of devices for drug screening and discovery and systems for tissue regeneration.

## Author Contributions

CC, LR, and MV gathered information and data from the literature and drafted the manuscript. MV and PN took the lead in organizing the final version of the manuscript. MV wrote the final version of the manuscript and edited figure panels. All authors provided critical feedback to shape the final version of the manuscript.

### Conflict of Interest Statement

The authors declare that the research was conducted in the absence of any commercial or financial relationships that could be construed as a potential conflict of interest.
